# Measuring Audience Engagement for Public Health Twitter Chats: Insights From #LiveFitNOLA

**DOI:** 10.2196/publichealth.7181

**Published:** 2017-06-08

**Authors:** Kristina M Rabarison, Merriah A Croston, Naomi K Englar, Connie L Bish, Shelbi M Flynn, Carolyn C Johnson

**Affiliations:** ^1^ Centers for Disease Control and Prevention Division of Population Health Atlanta, GA United States; ^2^ Tulane Univeristy School of Public Health and Tropical Medicine Tulane Prevention Research Center New Orleans, LA United States; ^3^ City of New Orleans Health Department New Orleans, LA United States

**Keywords:** social media, Twitter, Twitter chat, public health, communication, content analysis, social network analysis

## Abstract

**Background:**

Little empirical evidence exists on the effectiveness of using Twitter as a two-way communication tool for public health practice, such as Twitter chats.

**Objective:**

We analyzed whether Twitter chats facilitate engagement in two-way communications between public health entities and their audience. We also describe how to measure two-way communications, incoming and outgoing mentions, between users in a protocol using free and publicly available tools (Symplur, OpenRefine, and Gephi).

**Methods:**

We used a mixed-methods approach, social network analysis, and content analysis. The study population comprised individuals and organizations participating or who were mentioned in the first #LiveFitNOLA chat, during a 75-min period on March 5, 2015, from 12:00 PM to 1:15 PM Central Time. We assessed audience engagement in two-way communications with two metrics: engagement ratio and return on engagement (ROE).

**Results:**

The #LiveFitNOLA chat had 744 tweets and 66 participants with an average of 11 tweets per participant. The resulting network had 134 network members and 474 engagements. The engagement ratios and ROEs for the #LiveFitNOLA organizers were 1:1, 40% (13/32) (@TulanePRC) and 2:1, −40% (−25/63) (@FitNOLA). Content analysis showed information sharing (63.9%, 314/491) and health information (27.9%, 137/491) as the most salient theme and sub-theme, respectively.

**Conclusions:**

Our findings suggest Twitter chats facilitate audience engagement in two-way communications between public health entities and their audience. The #LiveFitNOLA organizers’ engagement ratios and ROEs indicated a moderate level of engagement with their audience. The practical significance of the engagement ratio and ROE depends on the audience, context, scope, scale, and goal of a Twitter chat or other organized hashtag-based communications on Twitter.

## Introduction

Approximately a quarter (23%) of American adult (age 18+) Internet users and a fifth (19%) of the entire American adult population use Twitter [1]. Twitter is a free social channel where registered individuals or organizations (ie, users) can share 140-character messages called tweets. There are 302 million active, monthly users and 500 million tweets posted per day [2]. Users are identified with usernames preceded by the “@” symbol. A retweet occurs when a user repeats another user’s tweet; sometimes, it is designated with “RT.” A hashtag is any word or phrase preceded by the pound symbol (#); it enables users to organize tweets by topics.

Public health practitioners use Twitter for disease surveillance [3-7], information dissemination [8-11], emergency response [12-16], and community building [17-21]. Evidence shows public health entities need to better harness Twitter’s potential as a two-way communication tool to establish relationships and increase audience engagement to improve the reach of their health promotion activities [22-24]. In response to this need, many public health practitioners now lead or participate in Twitter chats as part of their efforts to engage audiences in two-way communications. Currently, little empirical evidence exists on the effectiveness of using Twitter as a two-way communication tool for public health practice, and this warrants further research [24-30].

Commercial Twitter engagement measuring tools exist; many include metrics such as the number of “likes” and the click-through rate in their engagement metrics. However, they do not focus on the incoming and outgoing communications that occur when one user mentions another user in a tweet. In addition, they require a purchasing fee or a recurring membership fee.

Our primary aim was to analyze whether Twitter chats facilitate engagement in two-way communications between public health entities and their audience. We also describe how to measure this two-way communication, incoming and outgoing mentions, between users in a protocol using free and publicly available tools (Symplur [31], OpenRefine [32], and Gephi [33]). We chose the tools used in the protocol because they are user-friendly and do not require advanced analytical skills. The protocol’s target audiences are public health practitioners, such as health communication specialists and social media managers who are comfortable with point and click applications.

## Methods

### Study Design

We used a case study of one Twitter chat (#LiveFitNOLA) organized by Tulane Prevention Research Center (@TulanePRC), a university-based research and education center, and the City of New Orleans Health Department’s Fit NOLA Initiative (@FitNOLA). Twitter chats are interactive, organized, and curated communications on Twitter. They focus on a specific topic and take place at predesignated times.

With a mixed-method approach, social network analysis (SNA), and content analysis, we analyzed (1) whether Twitter chats facilitated audience engagement among public health entities and their audience and (2) whether functional themes were present in the engagements between Twitter chat participants.

### Definition of Engagement

Twitter defines engagement as the number of times a user interacted with a tweet, including clicks, retweets, replies, follows, likes, links, cards, hashtags, embedded media, username, profile photo, or tweet expansion [34]. Similarly, studies on health-related Twitter engagement define engagement as the number of user-mentions (retweets and replies), favorites, clicks, or detail expansions [28,29,35]. For the scope of our study, we define engagement in two-way communications as the number of incoming and outgoing mentions between users. A mention can be a direct mention, a retweet, or a reply. Assessing the number of incoming and outgoing mentions between public health entities and their audience during a Twitter chat is a first step in addressing the evidence gap on the effective use of Twitter as an audience engagement tool in two-way communications for public health entities.

### #LiveFitNOLA

@TulanePRC and @FitNOLA organize, host, and curate a monthly Twitter chat using the hashtag #LiveFitNOLA. The chat is focused on health and wellness in New Orleans. It is used to inform and engage participants in open discussions about the New Orleans’ culture of health. The target audiences for the #LiveFitNOLA chat are organizations and individuals in New Orleans interested in health and wellness. As hosts, @TulanePRC and @FitNOLA coordinated the chat plans and preparations, created visual promotions and tweets, and invited Twitter followers and community partners to participate.

The first #LiveFitNOLA Twitter chat occurred on March 5, 2015, between 12:00 PM and 1:00 PM Central Time (CT). *Health & Fitness Magazine* (@HealthFitMag), a Louisiana-based and operated magazine, participated as a guest host and determined the chat topic; approved all questions in advance; and invited their Twitter followers and community partners to participate. In addition, the Centers for Disease Control and Prevention’s National Center for Chronic Disease Prevention and Health Promotion (@CDCChronic) played a supporting role by participating as a subject matter expert. @CDCChronic provided evidence-based information on healthy living but did not intend to initiate or seek discussions with chat participants. The chat organizers selected the chat’s date and time (lunch hour on a weekday in New Orleans) after consulting other national organizations that host recurring, public health-related Twitter chats. Posting tweets during lunch hour toward the end of the week is a recommended best practice for social media scheduling, including Twitter chats [36-38].

### Data and Study Population

We collected the #LiveFitNOLA Twitter chat transcript from the Symplur Healthcare Hashtag Project [31]. Symplur tracks and archives tweets associated with registered health care-related hashtags. Symplur has a free and publicly available interface, where anyone can register and search for health care-related hashtags. To access hashtag-specific Twitter transcripts, Symplur can be queried for defined time frames. Using OpenRefine, we created a directed, relational dataset with source and target data points from the #LiveFitNOLA transcript by isolating the @username from tweet content and removing all other data elements. OpenRefine is compatible with both Windows and Mac Operating Systems, and is a publicly available data-cleaning tool [32].

For this study, we collected the #LiveFitNOLA chat transcript during a 75-min period on March 5, 2015, from 12:00 PM to 1:15 PM CT. This time frame included the hour-long #LiveFitNOLA chat and 15 min of residual conversations. Thus, our study population was composed of the individuals and organizations that participated and were mentioned in the #LiveFitNOLA chat during the previously defined time frame. We identified the chat participants’ geographic location based on self-disclosed location listed in user profiles. We categorized the #LiveFitNOLA chat participants’ locations as New Orleans, other US locations, and not available.

All information included in our analysis is secondary data obtained from publicly available Twitter data. The CDC Human Research Protection Office (HRPO) designated the research activity conducted in this case study as exempt—HRPO Exemption Determination for Protocol #6803 “#LiveFitNOLA: A dissemination and translation case study.” The Tulane Institutional Review Board determined this research activity does not constitute human subjects research—ID #15-857090U “#LiveFitNOLA: Monthly Twitter Chats – A Community Engagement Project.”

### Social Network Analysis (SNA)

We conducted an SNA to measure and visualize engagement in two-way communications between #LiveFitNOLA chat organizers and other chat participants. The SNA allowed us to create an engagement ratio of outgoing to incoming mentions among Twitter chat participants, and a return on engagement measure (ROE). The engagement ratio and ROE provide a simple way for public health practitioners to measure audience engagement on Twitter.

An SNA is the study of relationships between a connection’s source and its target within a specifically defined and bounded network [39,40]. We defined relationships among the #LiveFitNOLA participants as the engagement in incoming and outgoing communications occurring when one participant mentions another participant. Specifically, a connection occurred when a participant mentioned another user by including their @username in a tweet, whether by quoting a tweet, retweeting, or directly addressing another user. We conducted the SNA with Gephi, a free, publicly available, and interactive network visualization and exploration software. Gephi is compatible with both Windows and Mac Operating Systems [33].

In our study, SNA focused on simple network components and metrics, including node, edge, degree, in degree, out degree, and a number of communities. A node (ie, a network member) is an individual or an organization in a network; it is represented by a circle in a network map [39,40]. An edge (ie, a connection) is the relational tie between a source node and a target node; a line or an arrow between a source node and a target node represents an edge [39,40]. An arrow represents unidirectional communication between the two nodes, and points from the source node to the target node [39,40]. A line represents bidirectional communications between the source node and the target node [39,40]. The #LiveFitNOLA network was composed of chat participants and any other users mentioned during the chat. An edge occurs when a source participant mentioned a network member [39,40]. The total number of edges, independent of direction, is called degree [39,40]. “In degree” is the total number of incoming edges or incoming mentions for a #LiveFitNOLA network member [39,40]. For example, the @TulanePRC in degree is the total number of times other #LiveFitNOLA participants mentioned @TulanePRC in their tweets. “Out degree” is the total number of outgoing edges or outgoing mentions for a #LiveFitNOLA network member [39,40]. For example, the @TulanePRC out degree is the total number of times @TulanePRC mentioned other #LiveFitNOLA network members in their tweets. We did not use other SNA metrics such as network density and centrality measures, which are difficult to interpret because of the practical scope of this case study. Instead, we developed two metrics—an engagement ratio and an ROE measure—to assess two-way communications, incoming and outgoing mentions, between public health entities, and their audience on Twitter.

**Figure 1:**
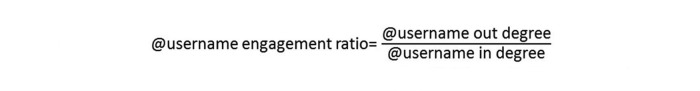
The engagement ratio equation.

The engagement ratio compares a network member’s total number of outgoing mentions (out degree) with their total number of incoming mentions (Figure 1, where, @username out degree is the number of outgoing mentions for a network member and @username in degree is the number of incoming mentions for a network member).

**Figure 2:**
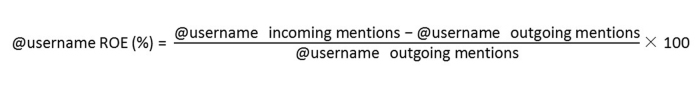
The return on engagement equation.

The ROE is based on the concept of return on investment (ROI), an investment performance measure. Like ROI does for monetary investments, the ROE measures the engagement gain or loss generated relative to the amount of engagement invested. In other words, the engagement gain or loss calculated as incoming mentions related to the number of outgoing mentions invested (Figure 2).

**Figure 3:**
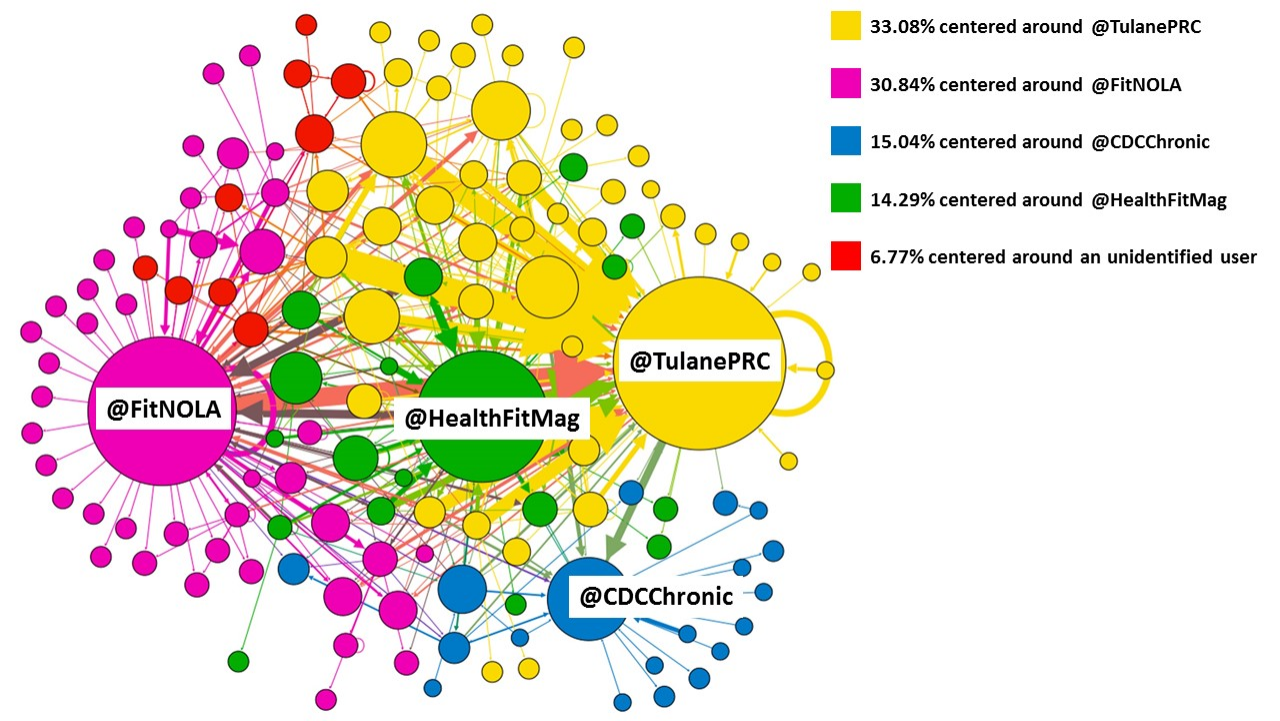
#LiveFitNOLA Twitter chat, March 5, 2015 from 12:00 PM to 1:15 PM Central Time, network map—Circles represent network members, which include #LiveFitNOLA chat participants and other Twitter users mentioned during the chat. Colors represent communities within the network; #LiveFitNOLA Twitter chat hosts are identified; other network members are not. Lines and arrows represent communications between network members. Lines are for two-way communications and arrows are for one-way communications.

To estimate the level of incoming and outgoing communications between public health entities and their audience, we focused our analysis on the #LiveFitNOLA Twitter chat organizers (@TulanePRC and @FitNOLA), the chat guest host (@HealthFitMag), and the supporting organization (@CDCChronic). We identified these usernames on the network map (Figure 3). Not all other participants were identified.

The step-by-step protocol for creating the SNA dataset is described in Multimedia Appendix 1. This protocol includes the Twitter chat transcript acquisition from Symplur, the transcript transformation into a relational dataset with source and target data points (or nodes) using OpenRefine, and network map visualization and measures with Gephi. An annotated and editable R code is in Multimedia Appendix 2, and can be used to collect hashtag-based tweet transcripts from Symplur. Public health professionals able to use point and click apps and with basic quantitative skills likely have the appropriate skills to conduct this analysis for organizations’ Twitter chats. The edits required for the provided R code in Multimedia Appendix 2 do not require R program language knowledge; instead edits are changes to the usernames of interests and transcript pages.

### Content Analysis

The content analysis assessed the primary function of tweets posted during the #LiveFitNOLA Twitter chat. We adapted a preexisting coding scheme originally developed by Lovejoy and Saxton to characterize tweets posted by public health entities [41]. They identified and described three primary themes: (1) information sharing, (2) community building, and (3) action- or activism-related [41]. Xu et al revised their coding scheme (herein: Information-Community-Action [I-C-A] framework) to characterize tweets that included health-related hashtags [41,42]. We applied a variant of Xu et al’s version [42] of the I-C-A framework to the #LiveFitNOLA Twitter chat transcript. First, we piloted their version of the I-C-A framework using a random sample of 50 original tweets posted during the chat. Based on the pilot, we altered the I-C-A framework themes, sub-themes, and definitions to include 3 themes and 4 subthemes: information sharing (health information, health opinion, health experience, and asking); positive affect or interpersonal closeness; action or activism or advocacy.

We applied the adjusted (I-P-A) coding scheme to a dataset of original tweets, which excluded all retweets to avoid biasing the distribution of themes and sub-themes. Of the 744 tweets in the full #LiveFitNOLA Twitter chat transcript, 491 (66%) were original tweets and 253 (34%) were retweets. Two analysts independently coded all original tweets for the most applicable theme and subtheme. They collaboratively reconciled coding disagreements resulting in percentages of agreement of 89% for themes and 74% for subthemes. We calculated inter-coder reliability for theme and subtheme using Cohen Kappa and Scott Pi. For theme, Cohen Kappa and Scott Pi were both .78. For subtheme, Cohen Kappa and Scott Pi were both .66.

## Results

### Data and Study Population

The #LiveFitNOLA Twitter chat had 744 tweets and 66 participants, with an average of 11 tweets per participant, during the 75-min study period. More than half of the #LiveFitNOLA chat participants were individual users (n=35, 53%). Twenty-six participants (39%) were organizational users, and 5 (8%) were uncategorized users. As expected, the majority (n=39, 60%) of the #LiveFitNOLA chat participants were from New Orleans. Fifteen (23%) were from other US locations, and 12 (18%) were from unidentified locations (Table 1).

### Social Network Analysis

The resulting #LiveFitNOLA network had 135 network members and 474 edges connections.

The network members included all 66 #LiveFitNOLA participants and 69 other Twitter users mentioned during the chat. Independent of direction, the overall number of connections (ie, outgoing and incoming mentions) per network member ranged from 1 to 101, with an average of 8 (SD=16) connections per network member. On average, there were 4 (SD=9) outgoing mentions per network number, and 4 (SD=7) incoming mentions per network member (Table 2).

The network had 5 distinct communities. More than half of the network’s connections formed 2 distinct communities, centered on the 2 #LiveFitNOLA chat organizers, @TulanePRC (33.3%) and @FitNOLA (31.8%) (Figure 3). Among all #LiveFitNOLA chat participants, @HealthFitMag (84) and @TulanePRC (82) tweeted the most. @TulanePRC had the most incoming mentions (in degree=45), and @FitNOLA had the most outgoing mentions (out degree=63; Table 3). The engagement ratios and ROEs for the #LiveFitNOLA organizers were 1:1 and 40% (13/32) (@TulanePRC) and 2:1 and −40% (−25/63) (@FitNOLA).

### Content Analysis

Table 4 shows the percentage of original tweets stratified by theme and sub-theme. Information sharing (63.9%, 314/291) and health information (27.9%, 137/491) were the most common theme and sub-theme, respectively. The second most common theme was positive affect or interpersonal closeness (31.7%, 156/491). The minority of original tweets was categorized as action or activism or advocacy (3.8%, 19/491) and unable to determine (0.4%, 2/491).

**Table 1:** March 5, 2015 (12:00 PM to 1:15 PM Central Time) #LiveFitNOLA Twitter chat participant characteristics.

Participant characteristics		Total number of participants, N=66 Total number of tweets, N=744
Average number of tweets per participant, mean (SD)		11 (17)
**Type of participant, n (%)**			
	Individual users	35 (53)
	Organizational users	26 (39)
	Uncategorized users	5 (8)
**Participant locations, n (%)**			
	New Orleans	39 (60)
	Other US locations	15 (23)
	Unavailable locations	12 (18)

**Table 2:** March 5, 2015 (12:00 PM to 1:15 PM Central Time) #LiveFitNOLA Twitter chat network characteristics.

Network characteristics	Total number of network members^a^, N=135 Total number of connections^b^, N=474
Degree^c^, mean (SD)	8 (16)
Out degree^d^, mean (SD)	4 (9)
In degree^e^, mean (SD)	4 (7)

^a^Network members include 66 #LiveFitNOLA Twitter chat participants and 69 Twitter users mentioned during the chat.

^b^Number of communications: The number of mentions during the #LiveFitNOLA Twitter chat ie, the total number of times a Twitter user mentions another user by including their @username in a tweet, whether by quoting a tweet, retweeting (repeating another user’s tweet), or directly addressing another user.

^c^Degree: Number of undirected communications between a source and a target Twitter user within the network regardless of the direction of the communication.

^d^Out degree: Number of outgoing communications a source Twitter user sent to other Twitter users within the network.

^e^In degree: Number of incoming communications a target Twitter user received from other Twitter users within the network.

**Table 3:** March 5, 2015 (12:00 PM to 1:15 PM Central Time) #LiveFitNOLA Twitter chat engagement ratios and return on engagement.

Usernames	Number of tweets	Outgoing^a^	Incoming^b^	Out:In^c^	ROE^d^
@TulanePRC	82	32	45	1:1	40%
@FitNOLA	70	63	38	2:1	−40%
@HealthFitMag	84	40	33	1:1	−18%
@CDCChronic	24	8	19	1:2	138%

^a^Outgoing: Number of outgoing communications a source Twitter user sent to other Twitter users within the network.

^b^Incoming: Number of incoming communications a target Twitter user received from other Twitter users within the network.

^c^Engagement ratio: Ratio of outgoing to incoming communications a Twitter user of interest has. The engagement ratio is rounded to the next integer.

^d^ROE: The engagement gain or loss generated relative to the amount of engagement invested.

**Table 4:** March 5, 2015 (12:00 PM to 1:15 PM Central Time) #LiveFitNOLA Twitter chat Information-Community-Action (I-C-A) Framework: Coding categories for communication themes, tweet exemplars, and percentage.

Theme	Subtheme	Definitions and examples	n (%)
Information sharing	Health information	Disseminating research findings, tip or advice, tools or resources, health news, and general information about health-related events *A2: Social support influences all aspects of health. Proper support has been shown to health increase physical activity* *. #LiveFitNOLA*	137 (27.9)
	Health opinion	Expressing original (not merely affirming another tweet or statement) opinions on health-related issues (unbacked by information or data included in the tweet) *@TulanePRC A3. I also think we do not treasure our health or bodies and so we do not treat them as gifts! #LiveFitNOLA*	90 (18.3)
	Health experience	Sharing personal or family or friend or experience with health problems or regarding health-related topics *@CDCChronic yep! I don’t go to a Gym. I do workouts on YouTube #LiveFitNOLA*	73 (14.8)
	Asking	Asking questions about health or health-related issues *@Tastedat is all about food so wheres your favorite place to eat out health? #LiveFitNOLA*	14 (2.8)
Positive affect or Interpersonal closeness		Showing positive affect such as appreciation, greeting, agreement, affirmation, and congratulation; showing interpersonal closeness *@frenchmktnola @tulaneprc so awesome! Thank you for joining! #LiveFitNOLA*	156 (31.7)
Action or activism or advocacy		Raising awareness, promoting health-related causes, and prompting receivers to take actions such as signing petition, making donation, sharing information, and participating in events *A3. Find out if you are at risk for diabetes and ways to prevent it bit.ly/1EORNDQ (2/2) #LiveFitNOLA*	19 (3.8)
Unable to determine		Lacks sufficient contextual information to determine the category or unrelated to health *@TulanePRC #LiveFitNOLA*	2 (0.4)

## Discussion

### Principal Findings

The trends in our findings indicate public health entities can use Twitter chats as a two-way communication audience engagement tool with their audience. To our knowledge, this is the first study that empirically examined Twitter audience engagement based on the incoming and outgoing communications between public health entities and their audience. To respond for the need to further assess the effectiveness of public health entities in two-way communications on Twitter, we developed metrics and a protocol to assess the incoming and outgoing communications between public health entities and their audience during a Twitter chat.

The #LiveFitNOLA chat organizers targeted New Orleans residents to engage in open discussions about the culture of health in the New Orleans area. A hashtag trends on Twitter when it is algorithmically determined to be one of the most popular hashtags or topics at a particular time [43]. Trends are hashtags or topics that are popular for a specific time and specific location. They are not the popular hashtags or topics that have been popular for a while or occur regularly [44]. Although trends are not representative of the public, they are representative of active Twitter users who are at a specific location during a specific time. A trending hashtag is a proxy measure of reach. For this particular chat, @TulanePRC and @FitNOLA reached members of the intended audience because #LiveFitNOLA was the sixth trending hashtag in New Orleans on March 5th, 2015 [45]. This was reflected in our findings, the majority of the #LiveFitNOLA chat participants were from New Orleans.

@TulanePRC was the main driver of this chat. Its engagement ratio revealed it received one incoming mention from a chat participant for each outgoing mention to another network member. Furthermore, @TulanePRC had a 40% ROE, which means it generated a 40% gain of incoming engagement related to the outgoing engagement it invested. @FitNOLA experienced a 40% engagement loss related to its invested engagement. By reading the chat transcript, we learned @FitNOLA retweeted most of @TulanePRC’s original tweets, which doubled its outgoing mentions compared with @TulanePRC’s outgoing mentions. @FitNOLA’s engagement level might be improved in the future if both #LiveFitNOLA organizers meet and coordinate to send original tweets rather than retweeting each other.

The guest host, @HealthFitMag, largely played a supporting role. It repeated most of the information shared by both #LiveFitNOLA organizers and did not provide original content. In future chats, the chat organizers should encourage guest hosts to share original content and actively respond to the chat participants. Interestingly, while playing a supporting role, @CDCChronic had the biggest return on its engagement at 138% and a 1:2 engagement ratio. @CDCChronic received twice as many incoming mentions as its outgoing mentions. Popular and influential Twitter accounts like @CDCChronic attract incoming communications from other users participating in hashtag and time-bound Twitter activities. As popularity begets attention, this might introduce false positives or false negatives to the engagement ratio and ROE. In this instance, #LiveFitNOLA chat participants sought to engage directly in two-way communications with @CDCChronic. @CDCChronic did not respond to the participants because its role was to disseminate evidence-based information on healthy living during the chat, not initiate or seek discussions like the Twitter chat hosts (@TulanePRC and @FitNOLA). The negative or positive popularity of certain users participating in hashtag-based Twitter interactions might influence how others will react to them; and in some cases, this is something beyond the control of the said user. For public health entities seeking to increase audience engagement in two-way communications, we recommend they address the roles of each account at the beginning of the chat. We also recommend taking into consideration the popularity or influence of certain accounts when interpreting the result of the engagement ratio and ROE.

Tweet sentiments might also influence the engagement ratio and ROE. For the scope of this case study, we did not conduct any sentiment analyses on tweet contents. We do recommend such analyses whenever possible to complement the engagement ratio and ROE results. An example of the importance of sentiment analyses would be the disclosure of a negative action, which would likely get a high ROE but might have highly negative reputational impact on the user. Mathematically, the ROE would be high but its impact on relationship building could be detrimental. Stylistic differences might influence the engagement ratio and ROE, and introduce false positives or false negatives because bombastic or offensive tweets might incite strong negative or positive reactions from other users. To this end, when interpreting the findings of the engagement ratio and ROE, we recommend skimming through the transcript to see if a user with a surprisingly low or surprisingly high engagement ratio and ROE have a very different tweeting style to other users.

The depth and breadth of incoming and outgoing communications between users might influence the interpretation of the engagement ratio. Depth represents how many times the same source mentions a specific target user (incoming) and how many times a specific source user mentions the same target user (outgoing). Breath measures mention volume; how many different users mention a specific target user (incoming), and how many different users mention a specific source user (outgoing). For example, of @TulanePRC’s 32 outgoing communications, 31 were sent to distinct different users and one was to itself, which indicates a broad-scale engagement with other users. In contrast, if a particular user had mentioned the same user 31 times, a single-source engagement would occur. If a single-source and a broad-scale engagement have the same engagement ratio and ROE, the engagement quality depends on the context. If the goal was to engage with more people such as in a Twitter chat, then a broad-scale engagement is better. If the goal was to engage in a one-on-one conversation, as might be the case for customer service interactions on Twitter, then a single-source engagement would be better.

Bots might interfere with the activities around a particular hashtag, and affect the engagement ratio and ROE with false positives. Tweets sent by bots need to be removed from the transcript before analyzing the incoming and outgoing engagement between users around a particular hashtag. The #LiveFitNOLA chat described here did not include any bot. However, the #LiveFitNOLA hashtag was hijacked by a bot in later periods of its use. We recommended the chat organizers to remove the bot’s activities from any analyses they might conduct within the period of the bot’s activities. In conclusion, user popularity, tweet sentiment and stylistic difference, and bots might influence the engagement ratio and ROE. Their practical significance depends on the audience, context, scope, scale, and goal of a Twitter chat or other organized hashtag-based communications on Twitter.

More advanced analyses could be conducted on networks formed on Twitter around hashtags. For example, in our case study, granular community-based analysis could be used to assess individual communities, cross-community, and within-community interactions. We did not conduct such analyses and did not include them in our protocol because they are beyond the practical purpose of our study. In addition, they require advanced knowledge of SNA beyond what is needed to obtain the engagement ratio and ROE. As previously mentioned, our intended audience are public health professionals able to use point and click apps and with basic quantitative skills.

In line with previous studies, results of the content analysis revealed the majority of tweets shared health information or showed positive affect or interpersonal closeness. The distribution of themes, however, deviated from prior research using the I-C-A framework [41,42]. Specifically, we identified more positive affect or interpersonal closeness tweets and fewer action or activism or advocacy tweets than other research that used the I-C-A framework [41,42]. These results highlight the potential of Twitter chats, which are typically prearranged, time-specific, moderated, and topically focused to enable participants cut through the millions of Tweets posted per day and establish directed connections. This might account for the increase in percentage of tweets that show positive affect or interpersonal closeness in our analysis, compared with other research using the I-C-A framework to analyze Twitter datasets that were not specific to a single Twitter chat.

### Limitations

A limitation for this case study is @FitNOLA and @HealthFitMag retweeted most of @TulanePRC’s tweets. Hosts retweeting hosts create an echo chamber and do not add new content, which might hinder host engagements with other users. Based on our findings, the hosts’ audience engagement might be improved by ensuring Twitter chats are organized and led by two or more organizations that do not retweet each other and individually tweet original contents. Each host should share carefully planned and curated original contents and refrain from sharing (retweeting) the same information as other hosts.

Another limitation is our use of self-disclosed geographical locations in user profiles. This is not an ideal way to identify geographic origins of tweets. We chose this approach because using the Global Positioning System (GPS) tags in tweets or Internet Protocol (IP) addresses would require more advanced analytical skills than the ones needed for the protocol we used in our study. In addition, users might not always enable their GPS-based location in their tweets, and IP addresses might not be accurate location indicators.

### Conclusions

Further studies are needed to establish quantifiable parameters on what is a low, medium, or high engagement ratio and ROE such as through longitudinal analyses of a recurring Twitter chat. In addition, such a study could provide more nuanced information about a public health entity’s Twitter audience for their hashtag-based communications such as their constituents’ health topic of interests or the type of guest host who will engage more with their audience.

The protocol described in Multimedia Appendix 1 on how to visualize and measure engagement levels for hashtag-based communications on Twitter is not limited to Twitter chats. It can be used for any time-bound hashtag-based Twitter communications where transcripts are available, such as conference hashtags (eg, #APHA15 for the 2015 American Public Health Association Annual Meeting), disease or condition hashtags (eg, #Ebola and #Zika), or public health initiatives or campaigns (eg, #CultureofHealth, a Robert Wood Johnson Foundation initiative to “enable all to live longer, healthier lives”).

Based on the lessons learned from this case study, to build further evidence on the effective use of Twitter as an audience engagement in two-way communication tool for public health entities our future research includes: (1) conducting longitudinal ROE assessments with recurring Twitter chats and (2) determining audience engagement effectiveness based on the hashtag-based communication’s purposes by examining larger public health campaigns.

## Appendix

Multimedia Appendix 1 How to visualize and measure engagement level for hashtag-based Twitter conversations.

Multimedia Appendix 2 R code to collect the #LiveFitNOLA chat transcript from Symplur, on March 5th 2015 from 1:00 PM to 2:15 PM ET, as an example (www.tinyurl.com/LiveFitNOLAMarch52015).

## Abbreviations

ROE: return of engagement
ROI: return on investment
SNA: social network analysis
