# Supplementary material for: Measuring Audience Engagement for Public Health Twitter Chats: Insights From #LiveFitNOLA
Source: JMIR Public Health Surveill. 2017 Jun 8;3(2):e34. doi: 10.2196/publichealth.7181 (PMC5481664; doi:10.2196/publichealth.7181)
Supplement: Multimedia Appendix 1 [file publichealth_v3i2e34_app1.pdf]

Measuring Twitter Chat Participant Engagement: #LiveFitNOLA Example

Appendix 1: How to visualize and measure engagement level for hashtag-based Twitter conversations

Kristina M. Rabarison, DrPH, MS

Merriah A. Croston, MPH

Naomi K. Englar, BA

Connie L. Bish, PhD, MPH

Shelbi M. Flynn, BA

Carolyn C. Johnson, PhD, FAAHB

Analytical Process

- Data source: Full 75-min transcript (744 tweets)
- Mapped participant engagement
  - 135 Twitter users (66 participants + 69 mentioned usernames)
  - 474 mentions (engagements between Twitter users)

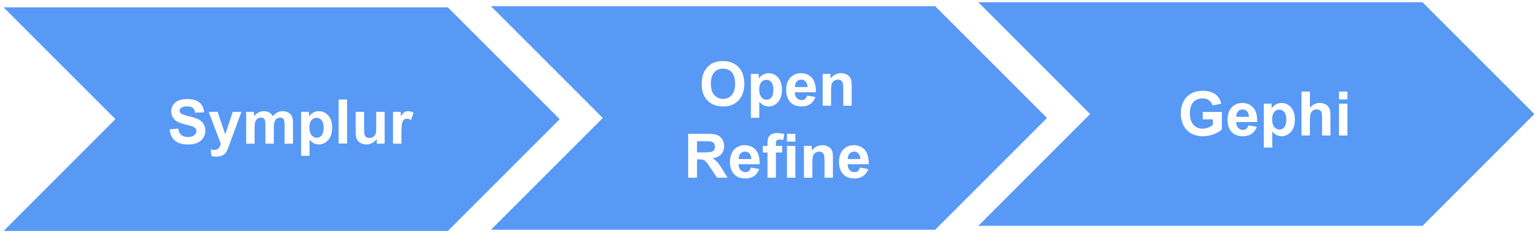

SYMPLUR

Register a Hashtag and Collect Twitter chat Transcript

How to submit a hashtag with Symplur:

Submit your hashtag The Healthcare Hashtag Project

Authenticate<br>Step 1	Select Hashtag Type<br>Step 2	Submit Information<br>Step 3	Get Confirmation<br>Step 4

You need to *Sign in with Twitter* before you can submit

Sign in with Twitter

Your hashtag should:

- be healthcare related
- be of value to the healthcare community
- be of a certain usage level by multiple people
- be unique enough that its intended conversation isn't drowned out by other uses of the same hashtag

How to collect hashtag-based Twitter two-way communication transcript from Symplur:

- Search for the hashtag of interest in Symplur (e.g. #LiveFitNOLA)

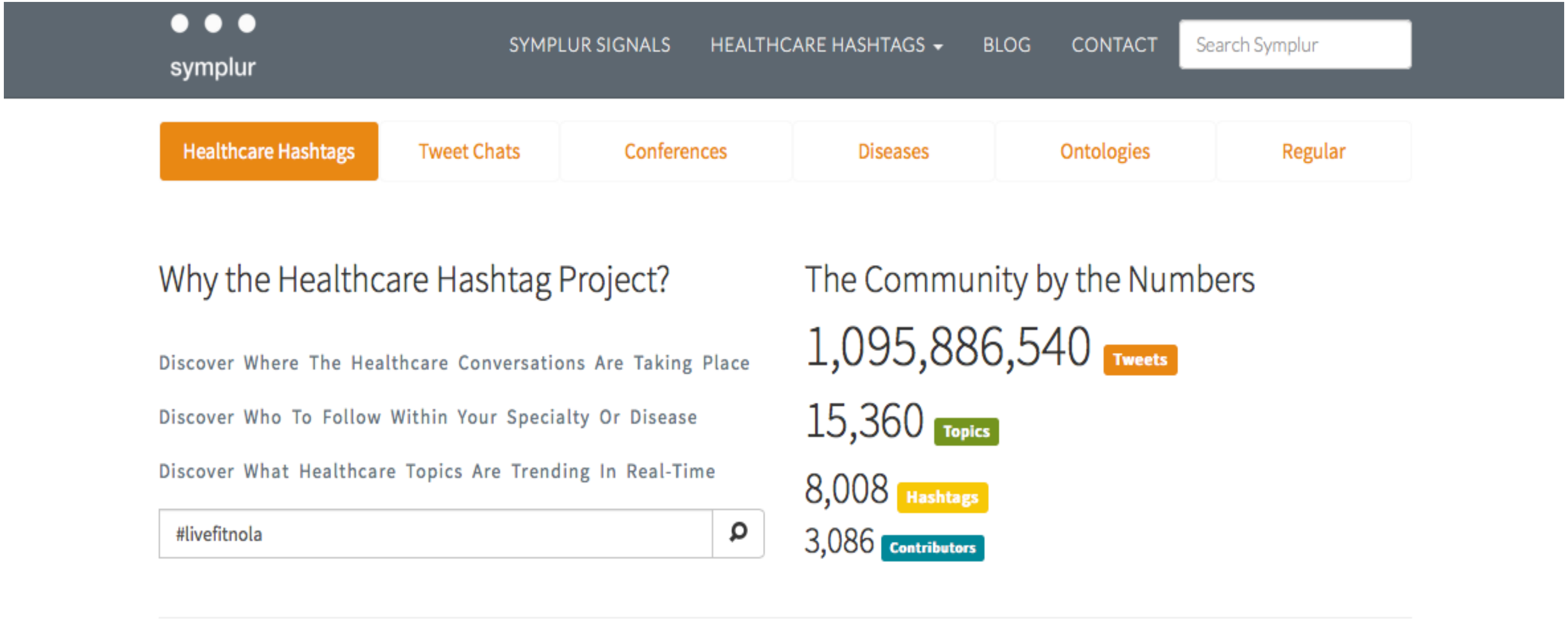

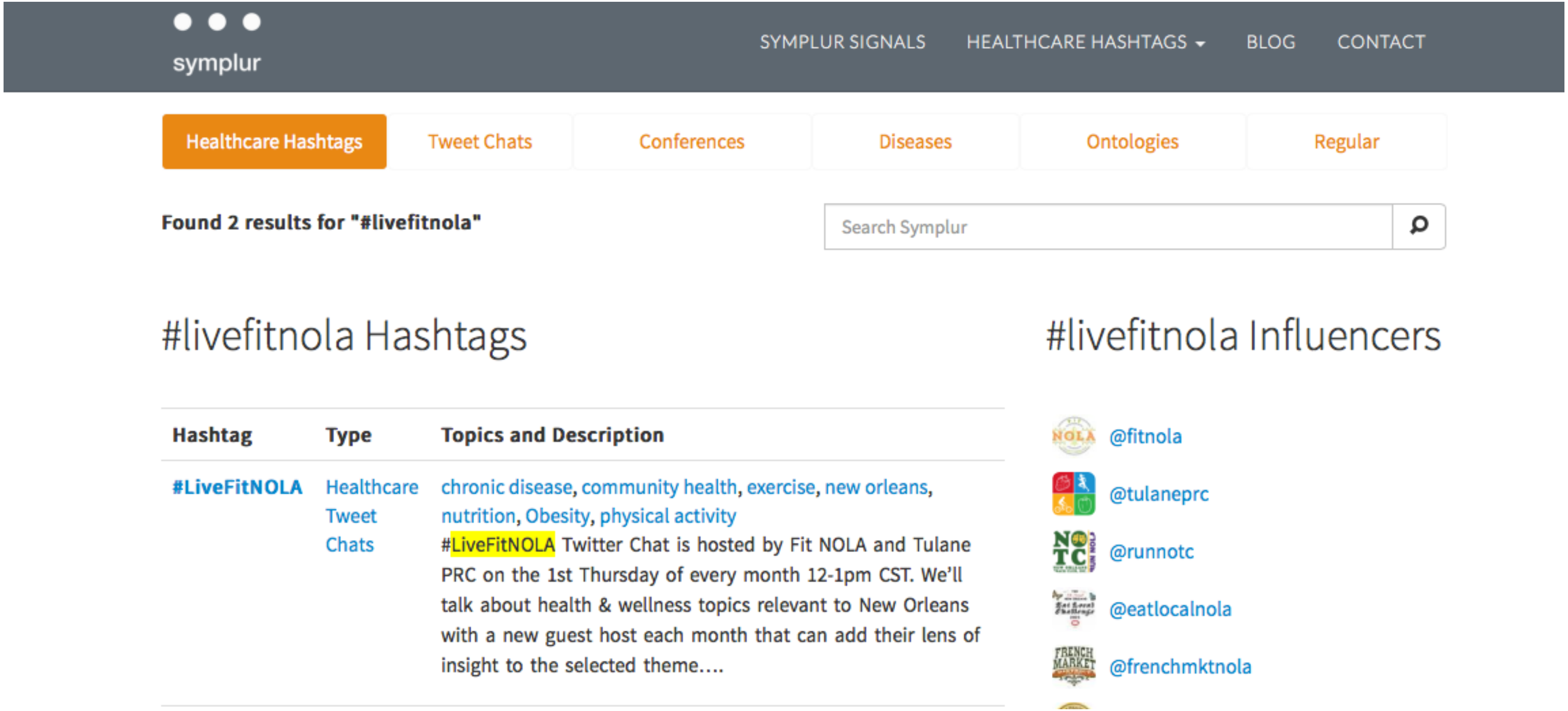
symplur
SYMPLUR SIGNALS
HEALTHCARE HASHTAGS
BLOG
CONTACT
Healthcare Hashtags
Tweet Chats
Conferences
Diseases
Ontologies
Regular
Found 2 results for "#livefitnola"
Search Symplur
#livefitnola Hashtags
#livefitnola Influencers
Hashtag
Type
Topics and Description
#LiveFitNOLA
Healthcare Tweet Chats
chronic disease, community health, exercise, new orleans, nutrition, Obesity, physical activity
#LiveFitNOLA Twitter Chat is hosted by Fit NOLA and Tulane PRC on the 1st Thursday of every month 12-1pm CST. We'll talk about health & wellness topics relevant to New Orleans with a new guest host each month that can add their lens of insight to the selected theme....
@fitnola
@tulaneprc
@runnotc
@eatlocalnola
@frenchmktnola

- Open hashtag page

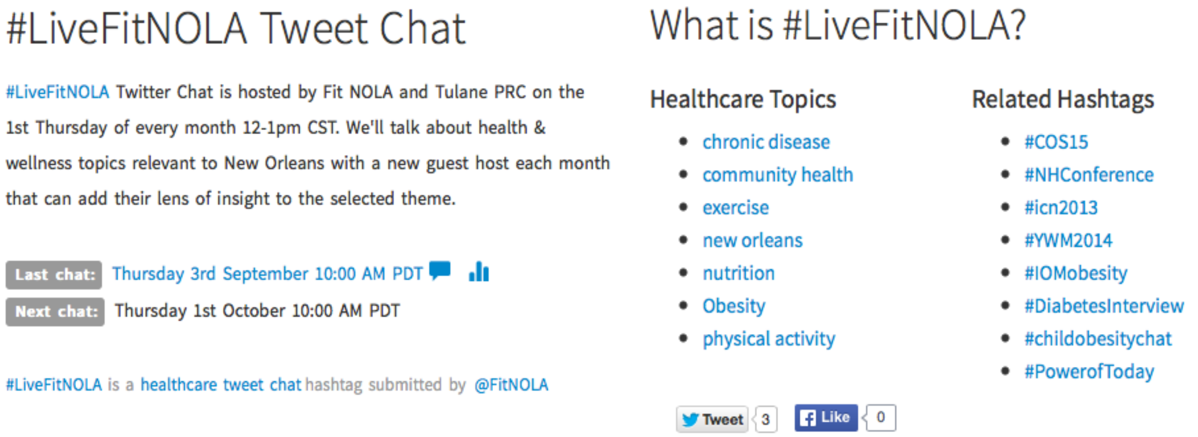

- Scroll down to the bottom of the page to set date and time

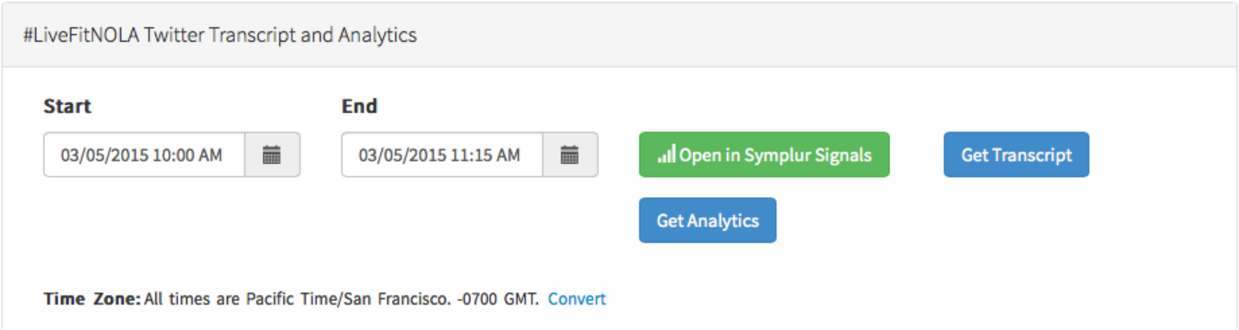

- Click on "Get Transcript" to load the transcript within the specifically defined timeline (Example transcript page: www.tinyurl.com/LiveFitNOLAMarch52015)
- Two options to collect data and time specific transcript:
  - Copy and paste the hashtag transcript from Symplur into an Excel file, saved as a .csv file.
  - Download R from www.r-project.org/. Then, use and edit the R code provided in Appendix B.

Open Refine

Prepare dataset for network visualization and analysis

How to format transcript data with OpenRefine:

- Download and install OpenRefine (www.openrefine.org/download.html)

Download OpenRefine

Home

Download

Documentation

Community

Post archive

OpenRefine News: Spring 2016

You will find on this page a list of OpenRefine distributions and extensions available for download. Are we missing something? Want to fix a typo? You can submit changes (pull request) from here.

Official Distribution

Read the installation instructions

OpenRefine 2.6

This is the first beta release of OpenRefine 2.6 on Aug 27, 2013. A change log is provided on the release page.

- **Windows kit**, Download, unzip, and double-click on *google-refine.exe*. If you're having issues with the above, try double-clicking on *refine.bat* instead.
- **Mac kit**, Download, open, drag icon into the Applications folder and double click on it.
- **Linux kit**, Download, extract, then type *./refine* to start.

- Launch OpenRefine and create a new project

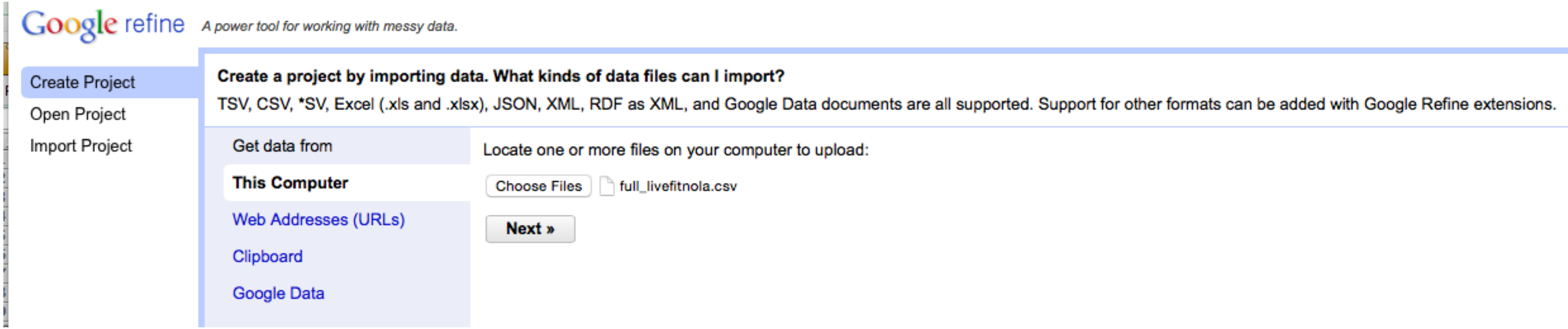

- Choose files from your computer and click on “Create Project” button

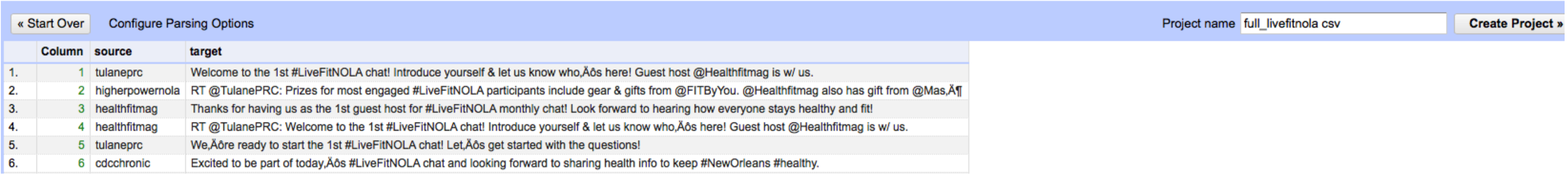

- Remove the first column, named "Column" and contains numbers

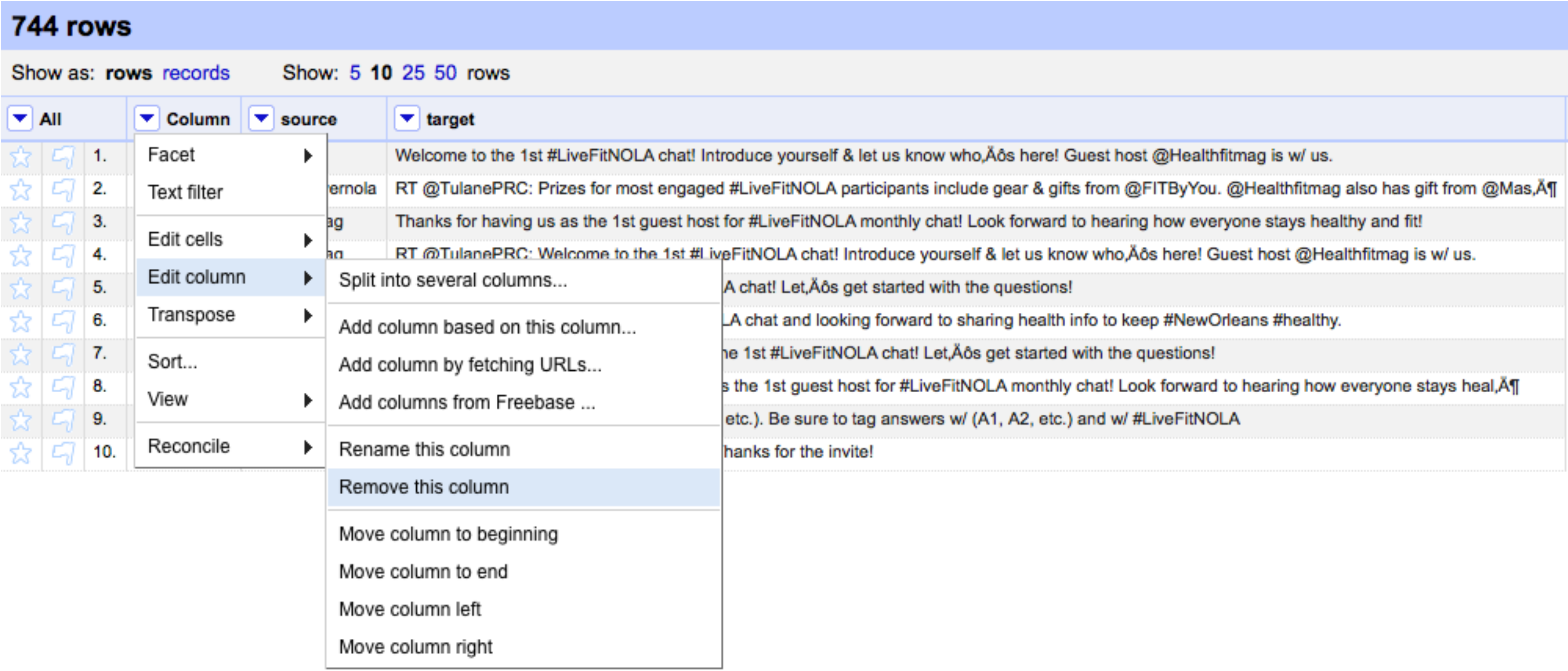

- Transform all the contents the “source” column to lowercase

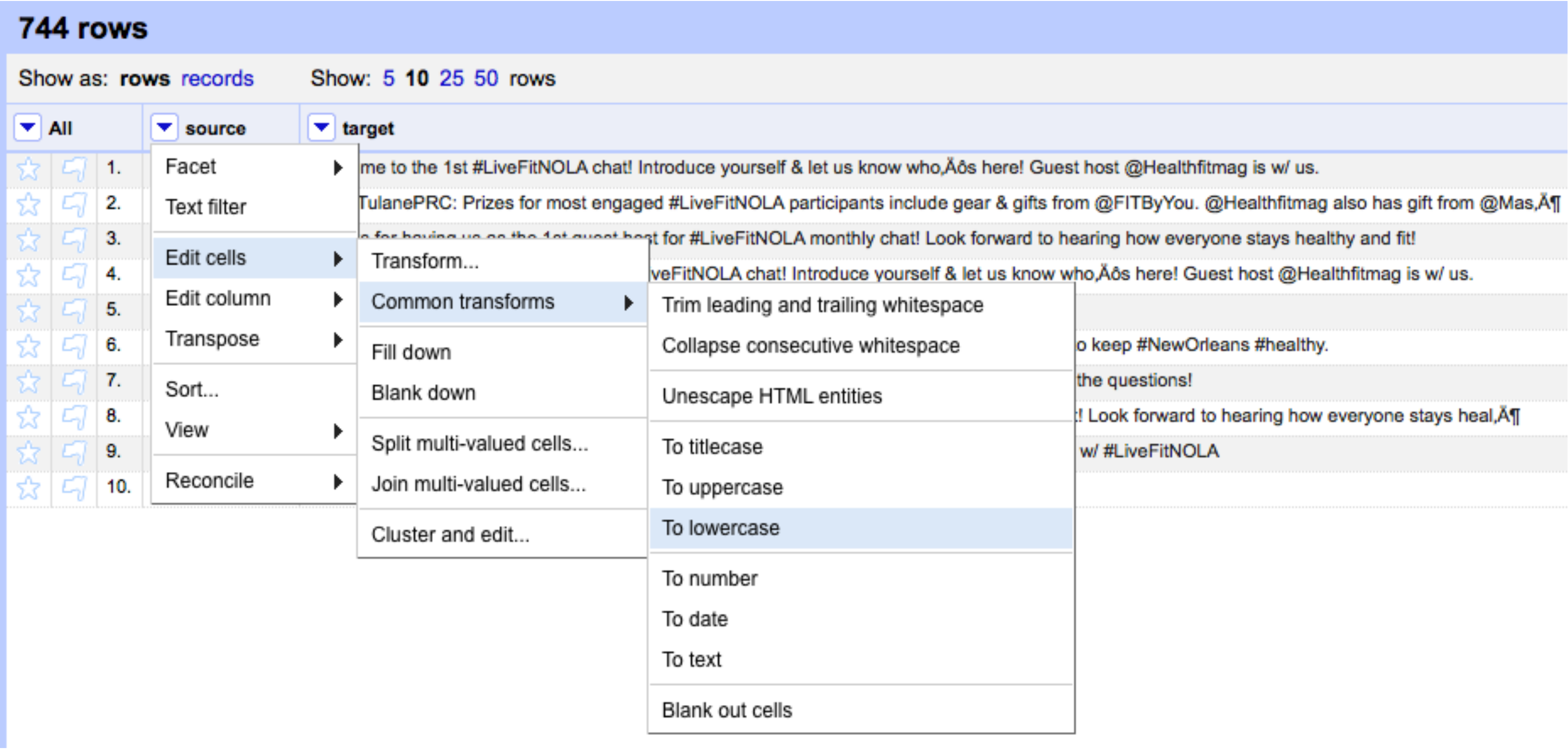

- Repeat the step above for the “target” column

- Transform all the contents the “source” column to be preceded by “@” symbol

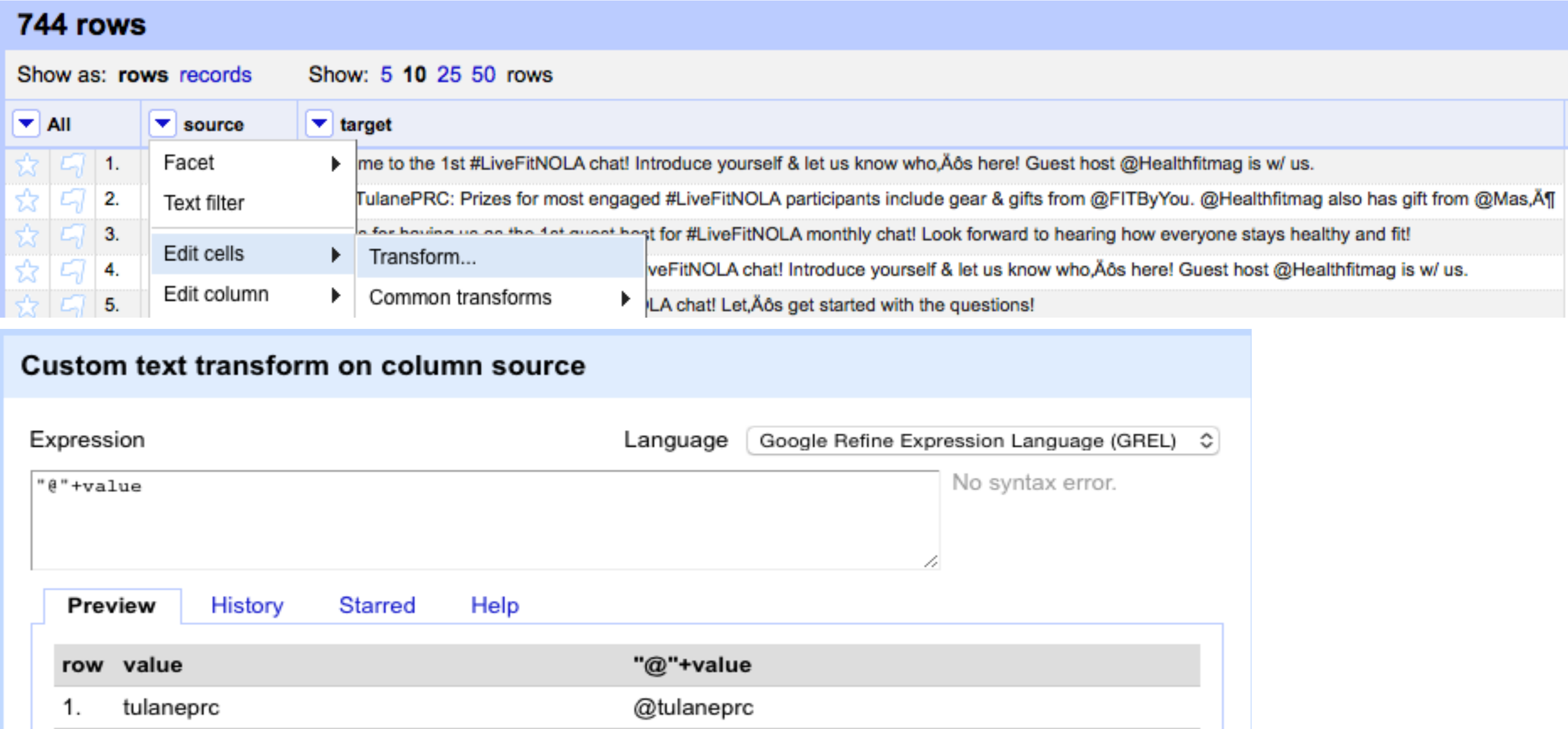

- Transform all the contents the “target” column
  - Copy: filter(value.split(/[^a-z0-9-_@#]/),i,(i.startsWith("@"))).join(",")
  - And paste in the Expression field below, to extract Twitter usernames mentioned in each tweet.

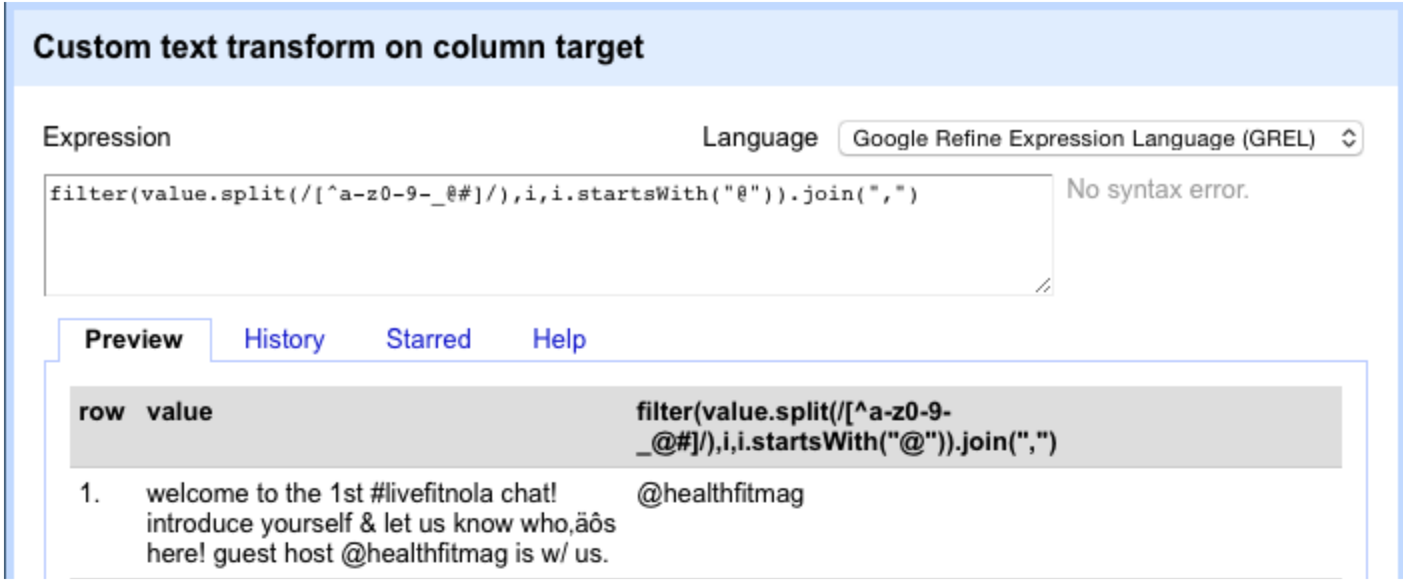

- Split the contents of the "target" column, to create set of source and target pairs

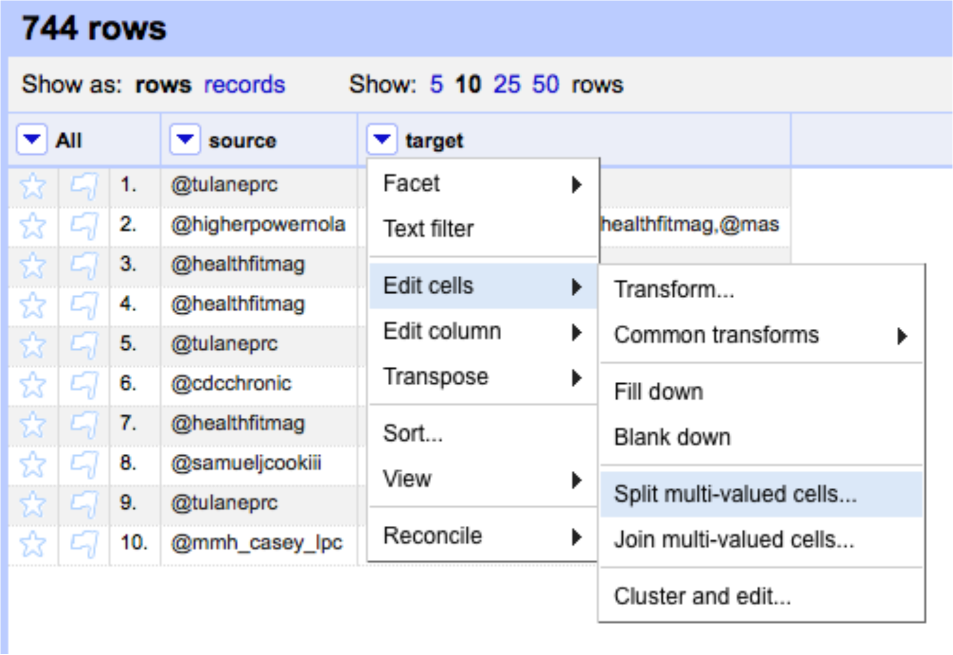

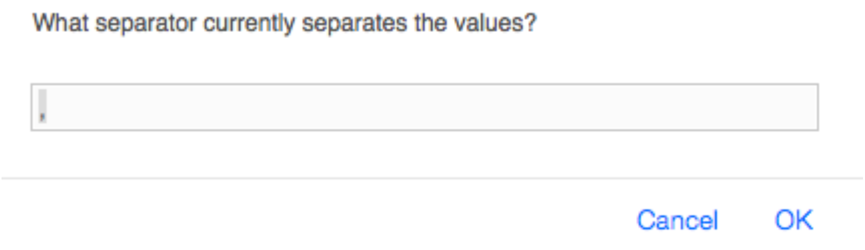

- Fill down the contents of the "source" column to finalize the relationship between source and target @usernames

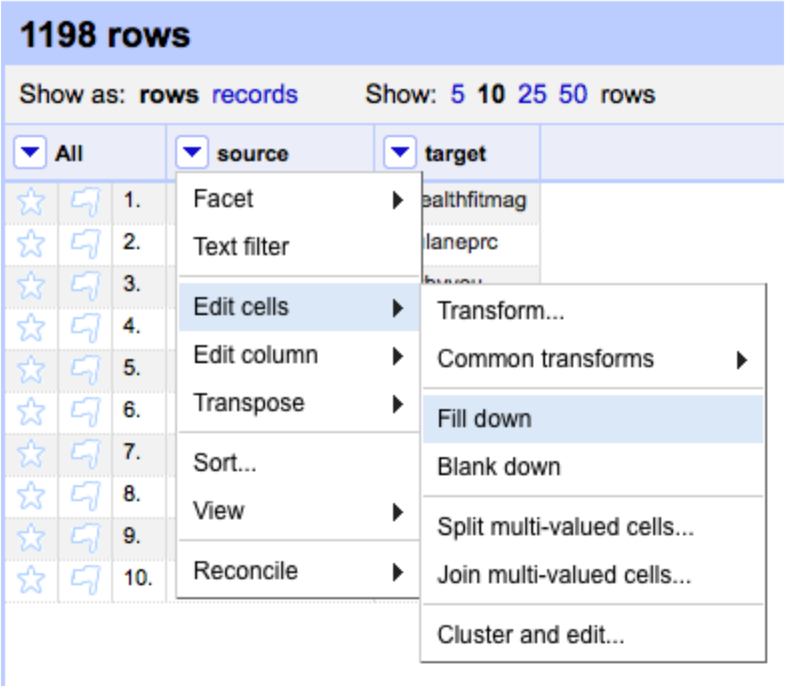

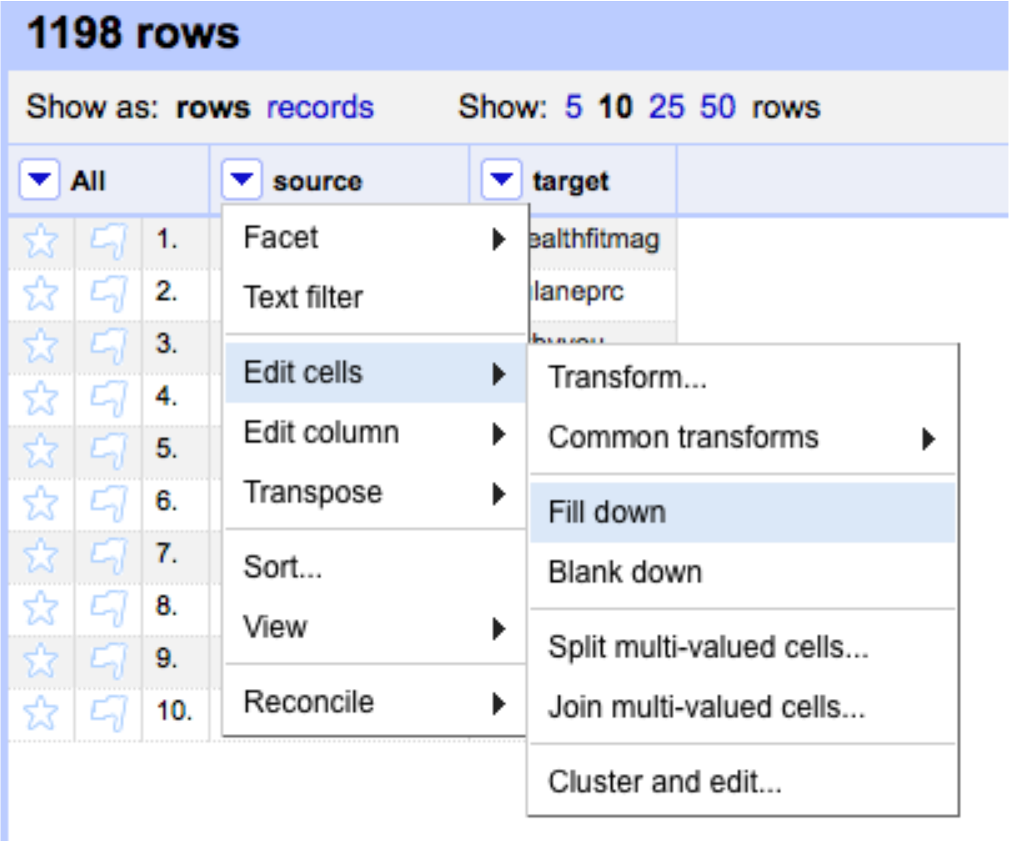
1198 rows
Show as: rows records
Show: 5 10 25 50 rows
All
source
target
1.
2.
3.
4.
5.
6.
7.
8.
9.
10.
ealthfitmag
laneprc
Facet
Text filter
Edit cells
Edit column
Transpose
Sort...
View
Reconcile
Transform...
Common transforms
Fill down
Blank down
Split multi-valued cells...
Join multi-valued cells...
Cluster and edit...

- Dataset for network visualization and analysis is now complete

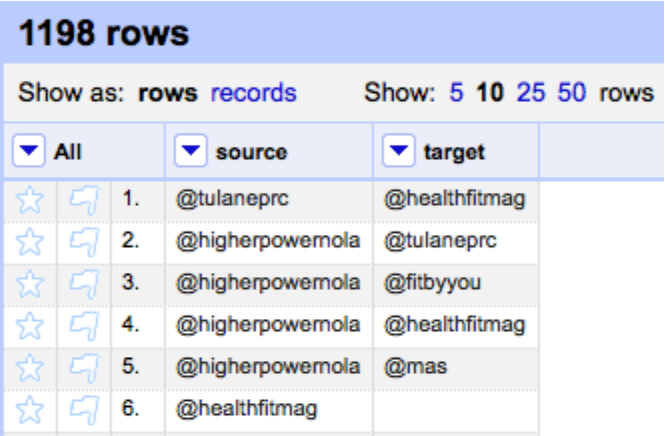

- Export the new dataset with the complete "source" and "target" columns as a .csv file

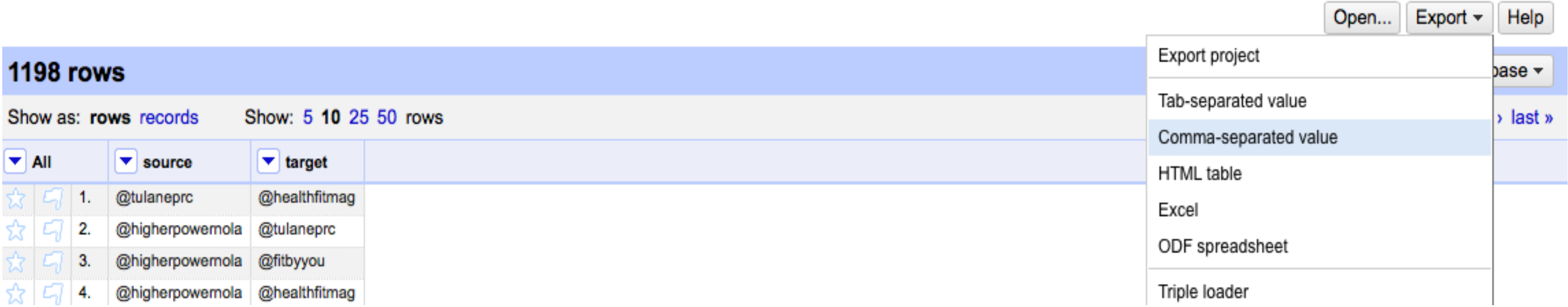

Gephi

Network mapping and analysis

How to visualize and collect a Hashtag-based two-way communication network with Gephi:

- Download Gephi (www.gephi.github.io/)

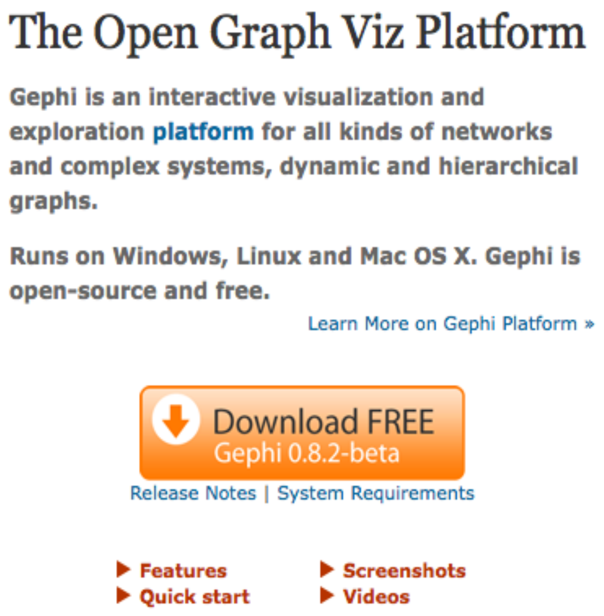

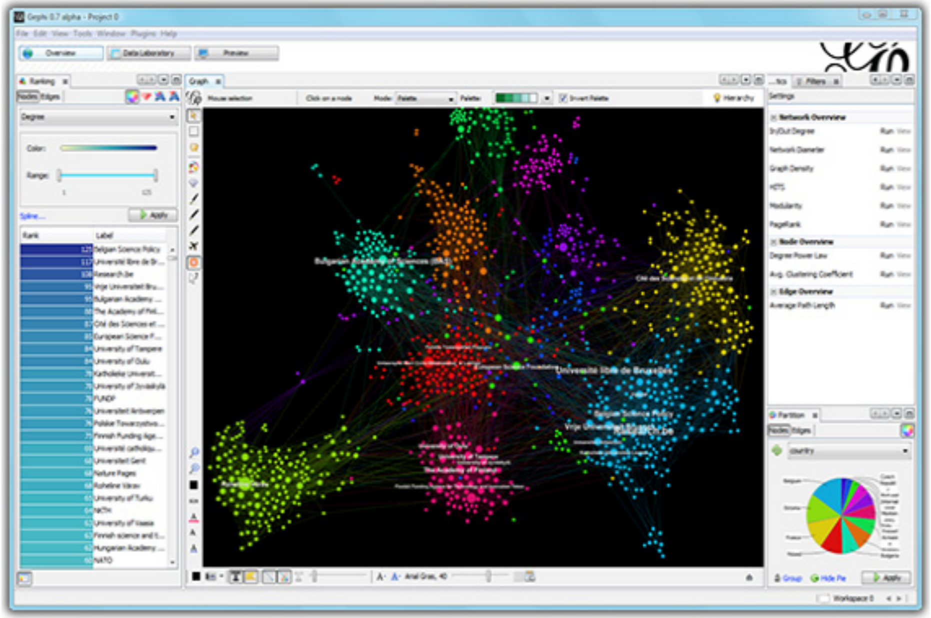

- Open Gephi and create a new project

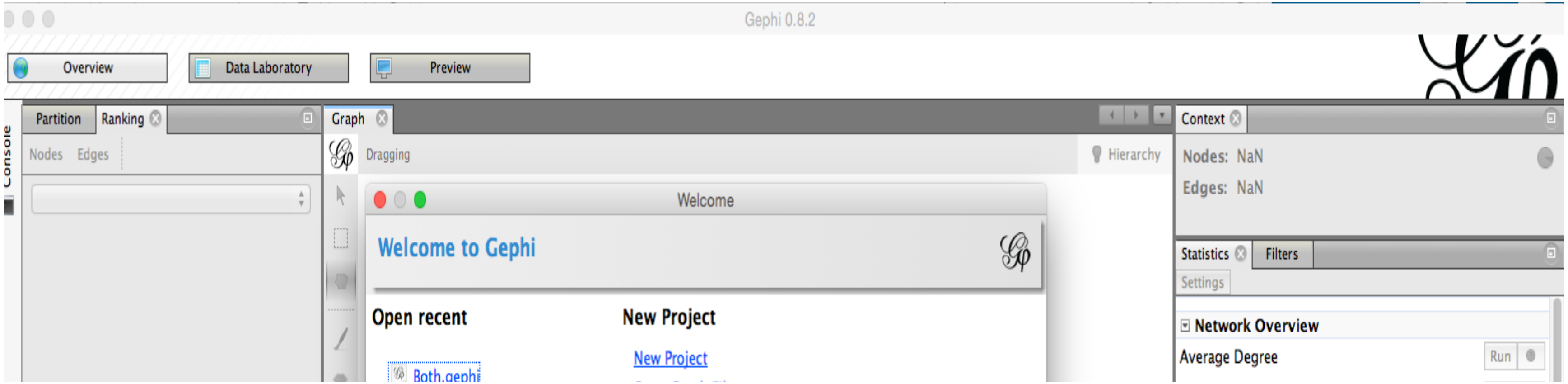

- Select the “Data Laboratory” then “Data Table”

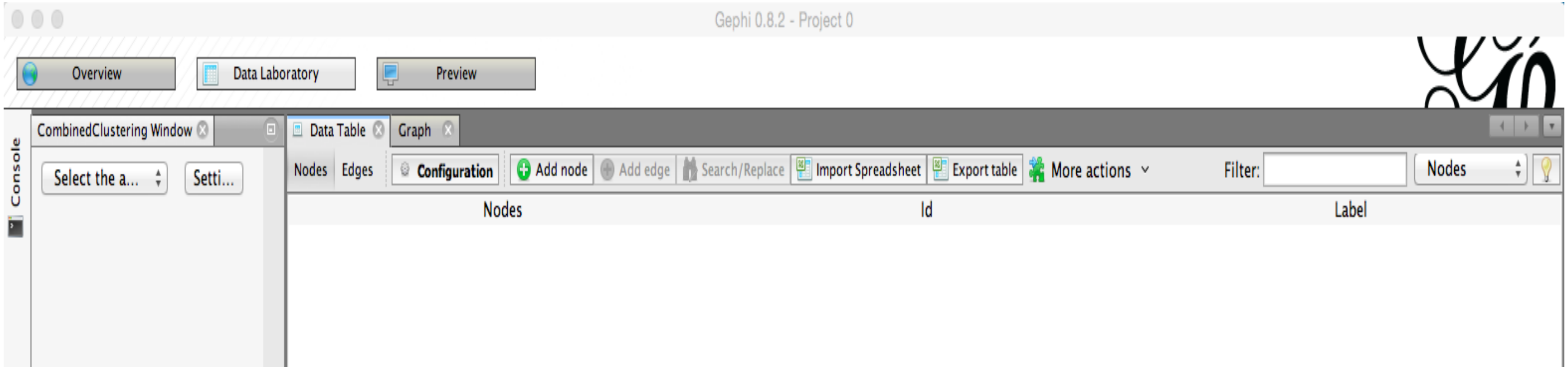

- Import Spreadsheet, the .csv file you saved at the end of OpenRefine step
  - Select “Comma” from the “Separator” drop down menu
  - Select “Edge table” from the “As table:” drop down menu

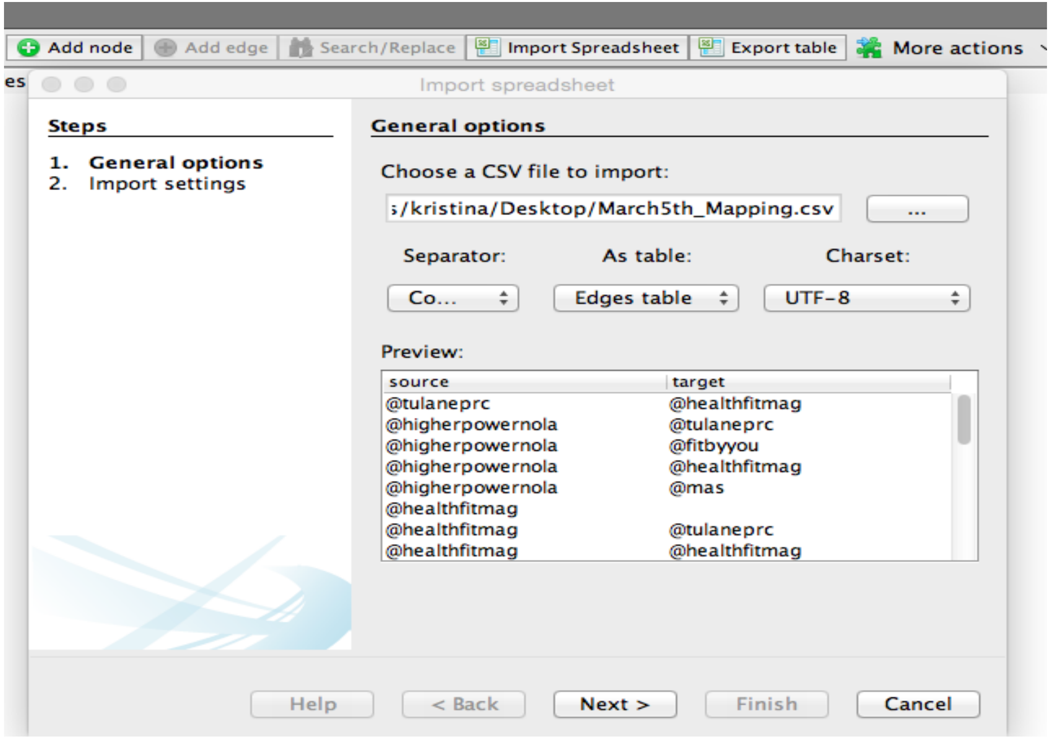

- Click the "Next" button in the import window
- And make sure you check the "Create missing nodes" option.

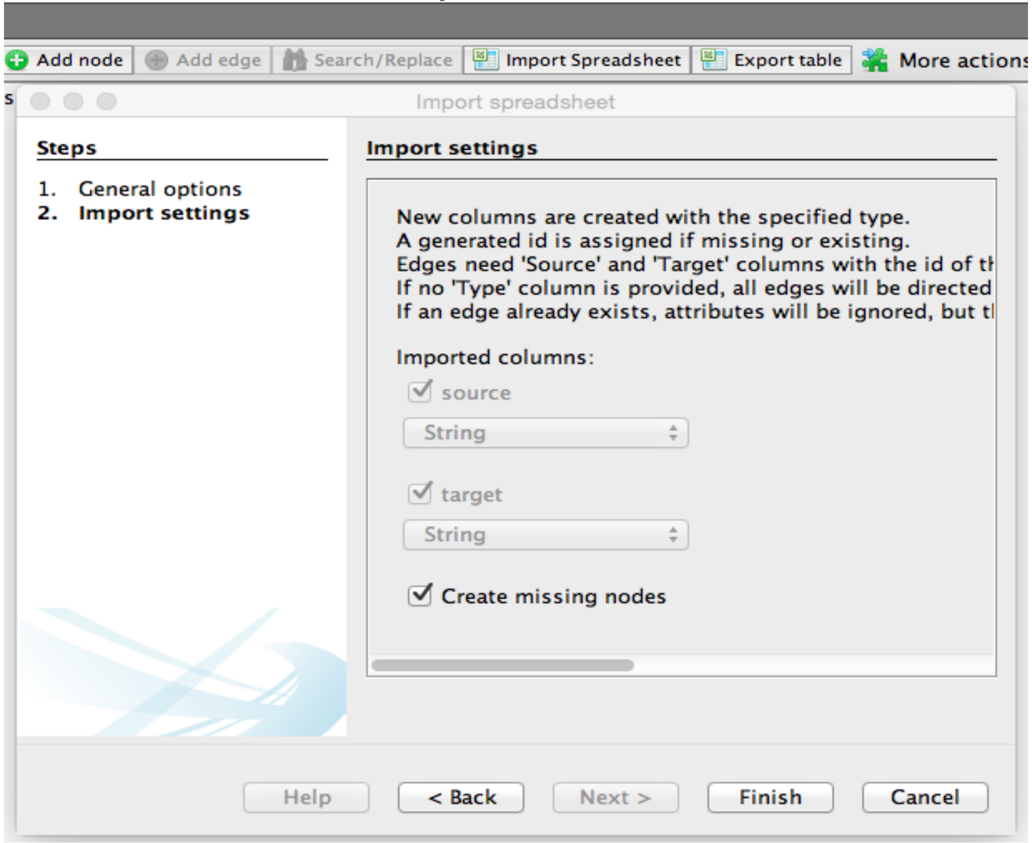

- Go back to the “Overview” tab and note how many “Nodes” and “Edges” are in the network. (Ex: Nodes: 135, Edges: 474).
  - Nodes represent the network members, which include the Twitter chat participants (here, 66 participants) and any other Twitter usernames they mentioned during the chat (here, 69 additional usernames mentioned).
  - Edges represent the relationships or connections between the nodes. In this case, edges are the connections formed when a #LiveFitNOLA chat participant mentioned another participants or another Twitter username.

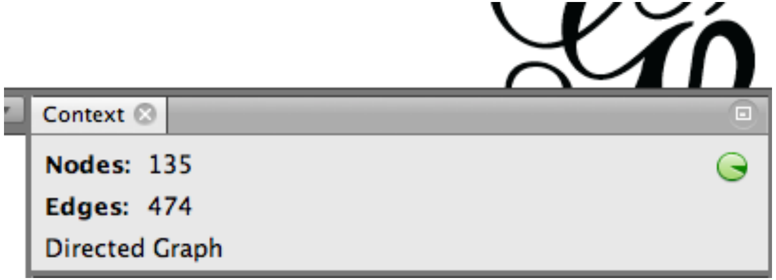

- Under the “Statistics” tab, run some simple metrics. For the purpose of this exercise, the two important metrics are: “Average Degree” and “Modularity”
  - Average degree is the average total number of mentions in the Twitter chat network, regardless of direction
  - Modularity is a community detection algorithm, which identifies the number of communities created in the Twitter chat network based on the number of engagements around particular nodes

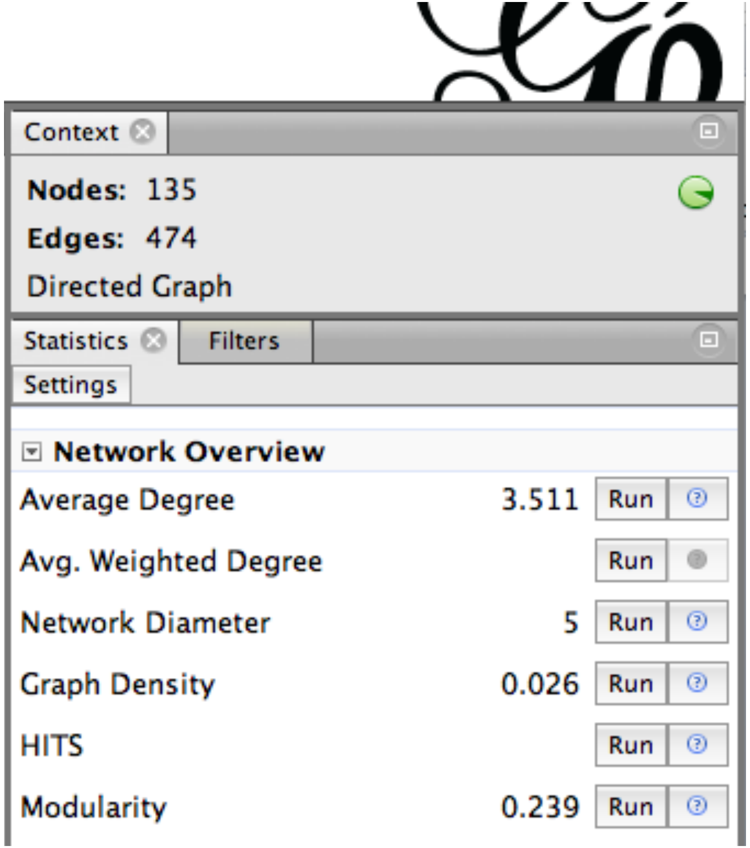

- Under the “Partition” and “Nodes” tabs, click on the green refresh arrows.
  - Choose “Modularity Class” as the partition parameter

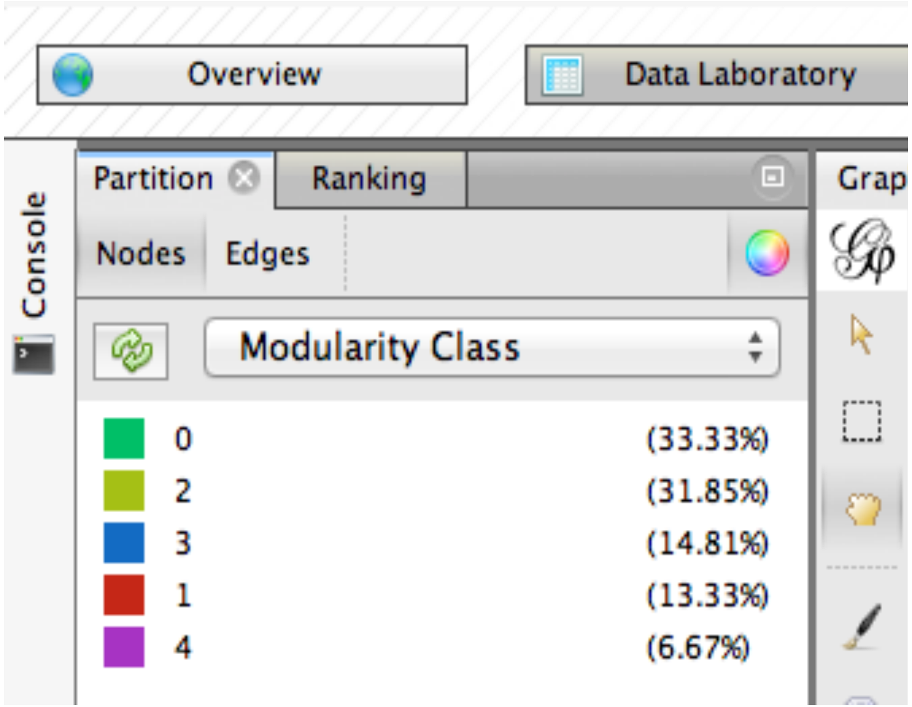

- Under the “Ranking” and Nodes tabs, choose “Degree” as the rank parameter.

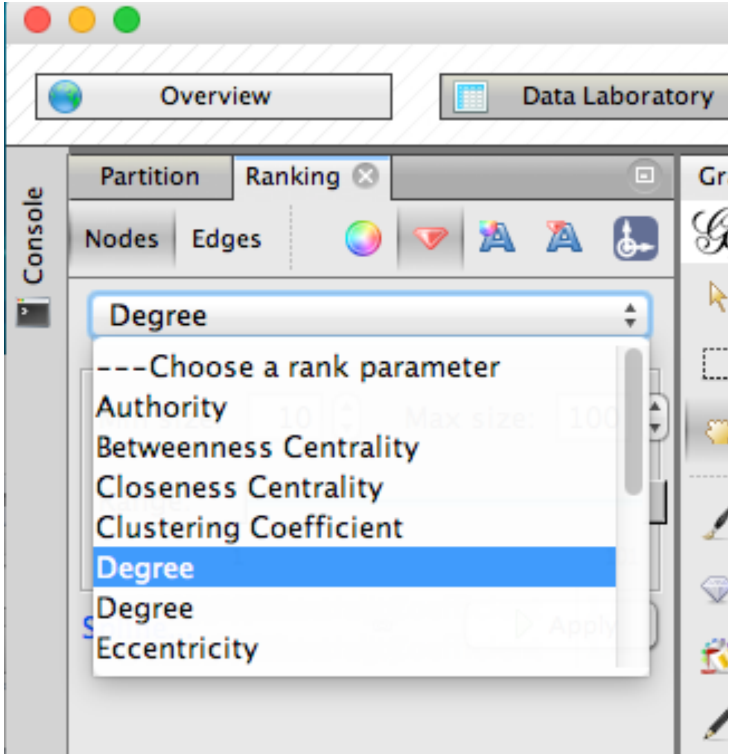

- Set the node sizes by clicking on the red diamond button, under the "Ranking" tab
  - e.g. min size = 10, max size = 100

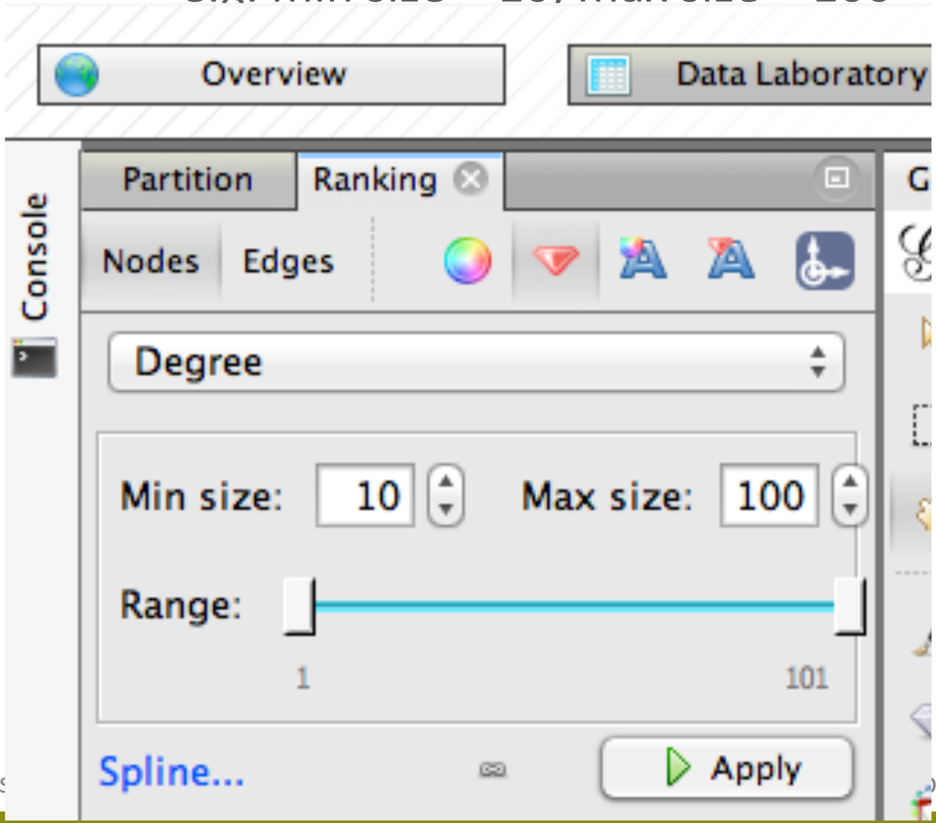

- Choose a layout to visualize the Twitter chat network map.
  - The layout choice is based on preference.
  - For the #LiveFitNOLA Twitter Chat, we first used the "Yifan Hu" layout, then "Noverlap" to remove the overlapping nodes in the network map.

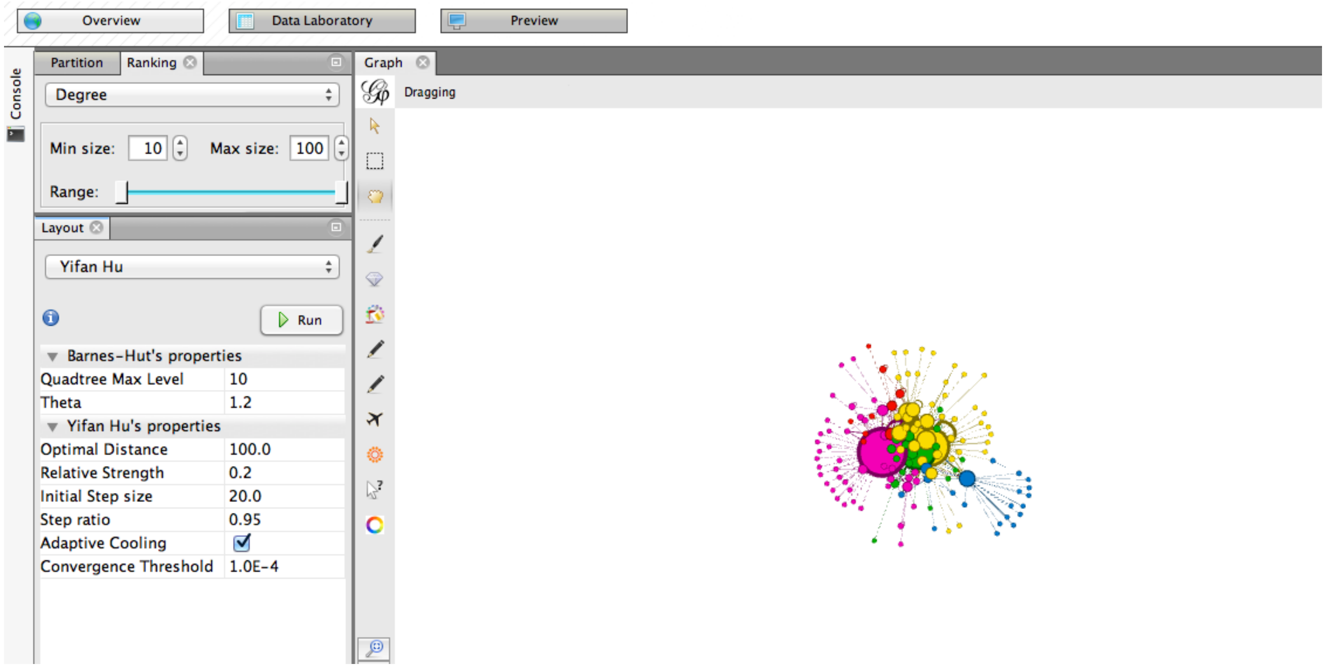

- Back to the “Data Laboratory” and “Data Table” tabs, export Nodes table and save.

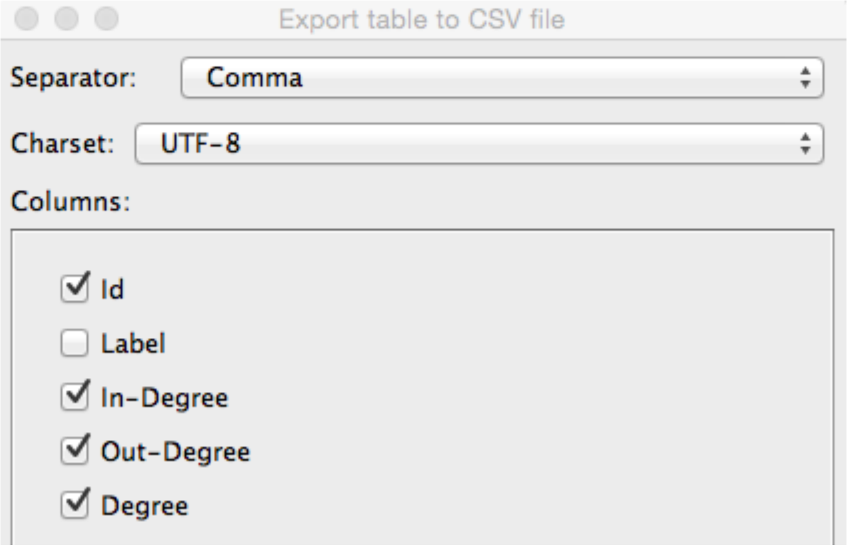

Network statistics:

- Import the Nodes table file you saved from the last step into Excel
- Run summary statistics for Degree, In Degree, Out Degree
- Identify your usernames of interest (e.g. @TulanePRC and @FitNOLA) and note the number of In Degree and Out Degree they had.
  - Number of in degree = number of incoming communications
  - Number of out degree = number of outgoing communications

#LiveFitNOLA Network

Analysis results

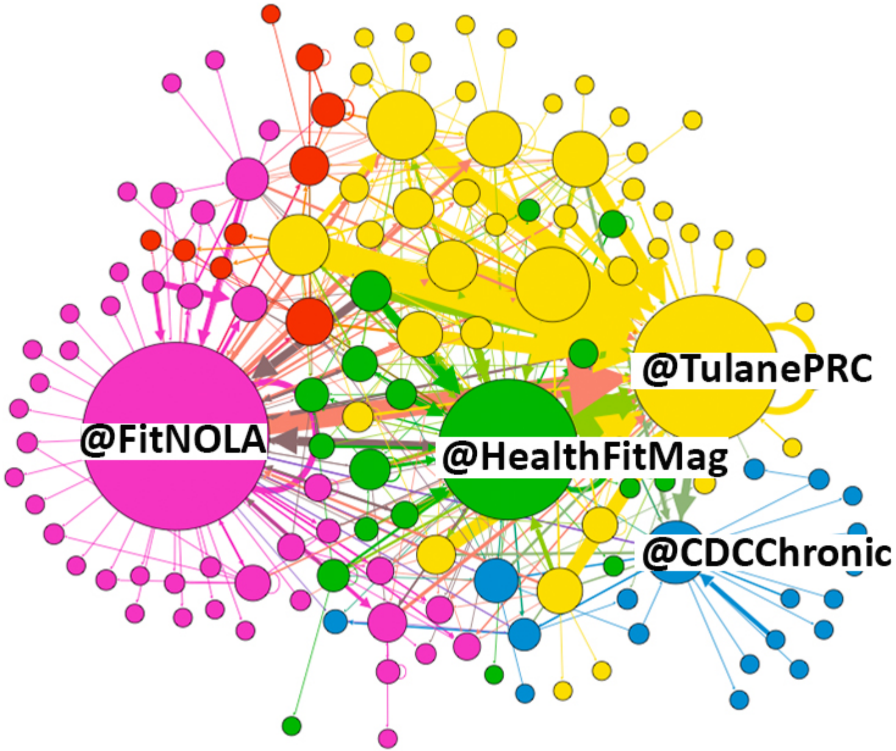

Total number of nodes = 135
Total number of edges = 474
Average network degree = 7 (13.4)
Degree range = 1 - 101

Number of communities = 5
- 33.33% centered around @TulanePRC
- 31.85% centered around @FitNOLA
- 13.33% centered around @HealthFitMag
- 14.81% centered around @CDCChronic
- 6.67% centered around an unidentified user

Definition of engagement:

- **Engagement** in two-way communications on Twitter is defined as the number of incoming and outgoing mentions between users.
  - A mention can be a direct mention, a retweet, or a reply
  - In other words the incoming and outgoing interactions between two Twitter users within a network bound by a hashtag and a specific timeframe.
- **Overall engagement** for a Twitter user of interest (ex: @TulanePRC) is the total number of two-way communications that user was involved in, regardless of direction.
- **Outgoing mention** is the number of times a Twitter user of interest mentions another user in a tweet.
  - For example: @TulanePRC's outgoing communication is **the total number of times @TulanePRC retweeted or replied to another Twitter user.**
- **Incoming mention** is the number times a Twitter user of interest was mentioned in another user's tweet.
  - For example: @TulanePRC's incoming communication is **the total number of times @TulanePRC other Twitter users retweeted or replied to @TulanePRC.**

Engagement ratio:

- **Engagement ratio** compares a network member's total number of outgoing mentions to their total number of incoming mentions.

$$\text{@username Engagement ratio} = \frac{\text{@username out degree}}{\text{@username in degree}}$$

@username out degree: The number of outgoing mentions for a network member.

@username in degree: The number of incoming mentions for a network member.

Return on engagement (ROE)

- **ROE** measures the engagement gain or loss generated relative to the amount of engagement invested.
  - In other words, the engagement gain or loss calculated as incoming mentions related to amount of outgoing mentions invested

$$\text{@username ROE (\%)} = \frac{\text{@username's total incoming mentions} - \text{@username's total outgoing mentions}}{\text{@username's total outgoing mentions}} \times 100$$

#LiveFitNOLA engagement levels:

	Outgoing	Incoming	Out:In	ROE
@TualnePRC	32	45	1 to 1	40%
@FitNOLA	63	38	2 to 1	- 40%
@HealthFitMag	40	33	1 to 1	-18%
@CDCChronic	8	19	1 to 2	138%

Thank You!

For questions or comments:

Kristina M. Rabarison, DrPH, MS
KRabarison@cdc.gov

For more information, contact CDC
1-800-CDC-INFO (232-4636)
TTY: 1-888-232-6348 www.cdc.gov

The findings and conclusions in this report are those of the authors and do not necessarily represent the official position of the Centers for Disease Control and Prevention.
