# Supplementary material for: Measuring Audience Engagement for Public Health Twitter Chats: Insights From #LiveFitNOLA
Source: JMIR Public Health Surveill. 2017 Jun 8;3(2):e34. doi: 10.2196/publichealth.7181 (PMC5481664; doi:10.2196/publichealth.7181)
Supplement: Multimedia Appendix 2 [file publichealth_v3i2e34_app2.pdf]

Appendix 2: R code to collect the #LiveFitNOLA chat transcript from Symplur, on March 5th 2015 from 1:00 PM to 2:15 PM ET, as an example (www.tinyurl.com/LiveFitNOLAMarch52015)

This appendix was developed to extract Twitter chat transcript, to replicate the analysis described in: Measuring audience engagement for public health Twitter chats: Insights from #LiveFitNOLA, KM Rabarison et al. 2016.

1. Download R from: https://www.r-project.org.
2. Copy and paste the R code below into a new R Script.

```
#Jennifer L. Kline, MPH (ORISE Fellow, CDC - Prevention Research
 Centers Program) wrote and developed this R code. The purpose of
 this R code is to extract Twitter usernames and tweet contents
 from a Symplur Twitter chat transcript or any other hashtag-
 based Symplur Twitter transcripts. As an example, this R code
 uses the transcript from the first #LiveFitNOLA Twitter chat, on
 March 5th 2015. To access the #LiveFitNOLA transcript, go to:
 www.tinyurl.com/LiveFitNOLAMarch52015.

#To replicate the #LiveFitNOLA Twitter username and tweet
 contents extraction using the R code below, replace the
 transcript URL (see URLbase) with your Twitter chat's URL and
 specify the number of URL pages your transcript has (see
 URLpage).

#BEGIN: To extract the #LiveFitNOLA transcript, copy and paste
 the code below into a new R Script.

#Install the following packages:

install.packages("XML")
install.packages("plyr")
install.packages("stringr")
install.packages("data.table")

#Load the packages:

library(XML)
library(plyr)
library(stringr)
library(data.table)

#For more information about these packages, see their Reference
 Manuals:
#XML: https://cran.r-project.org/web/packages/XML/XML.pdf
```

```
#plyr: https://cran.r-project.org/web/packages/plyr/plyr.pdf
#stringr: https://cran.r-project.org/web/packages/stringr/stringr.pdf
#data.table: https://cran.r-project.org/web/packages/data.table/data.table.pdf
#Set the working directory (e.g. a desktop folder called "R" containing individual project folders).
#Alternatively, set the working directory by going to the R menu, then selecting: "File", "Change Directory", and choosing a location.

setwd("~/Desktop/R/LiveFitNOLA")

#Set the transcript URL base. The base of the transcript URL contains page number information but not the actual page number.
#The #LiveFitNOLA transcript has two pages; go to the second page to get the transcript URL base.
#This is page 2 of the #LiveFitNOLA Transcript URL; the highlighted portion is the URLbase:
http://embed.symplur.com/twitter/transcript?hashtag=LiveFitNOLA&fdate=03%2F05%2F2015&shour=10&smin=00&tdate=03%2F05%2F2015&thour=11&tmin=15&page=2

URLbase <- 'http://embed.symplur.com/twitter/transcript?hashtag=LiveFitNOLA&fdate=03%2F05%2F2015&shour=10&smin=00&tdate=03%2F05%2F2015&thour=11&tmin=15&page='

#The LiveFitNOLA transcript includes 2 URL pages.
#If the transcript of interest only has 1 page, use "1:1" in the code below. If the transcript of interest has more than 2 pages, replace "2" in the code below with the total number of pages.

URLpage <- 1:2

#Combine the URL base with each of the page numbers and make a list of all transcript URLs.

URLs <- paste0(URLbase, URLpage)

#Extract data from each page of the transcript and organize the data into two columns:"Source": contains Twitter usernames; and "Target": contains tweet contents.
#These columns must be named Source and Target when imported into OpenRefine.

table <- list()
```

```
for (i in seq_along(URLs)){
  URLextract <- readHTMLTable(URLs[i], header=F)
  n.rows <- unlist(lapply(URLextract, function(t) dim(t)[1]))
  table[[i]] <- as.data.frame(URLextract[[which.max(n.rows)]])
}
TableData <- rbindlist(table)
TableData <- rename(TableData, c("V1"="Source", "V3"="Target"))
TableData <- subset(TableData, select=c("Source","Target"))
TableData$Target <- str_replace_all(TableData$Target, "\\n", "")
TableData$Target <- str_replace_all(TableData$Target, "Thu.*",
 "")
TableData$Target <- sub("^\\s+|\\s+$", "", TableData$Target)

#Export the data as a .csv file (e.g. "LiveFitNOLA.csv").
#This will be saved in the working directory.

write.csv(TableData, file = "LiveFitNOLA.csv")

#Import this .csv file into OpenRefine; see instructions in
 Appendix A.
```
